# An automated DIY framework for experimental evolution of *Pseudomonas putida*

**DOI:** 10.1111/1751-7915.13678

**Published:** 2020-10-13

**Authors:** David R. Espeso, Pavel Dvořák, Tomás Aparicio, Víctor de Lorenzo

**Affiliations:** ^1^ Systems Biology Program Centro Nacional de Biotecnología‐CSIC Campus de Cantoblanco Madrid 28049 Spain; ^2^ Department of Experimental Biology Faculty of Science Masaryk University Brno 62500 Czech Republic

## Abstract

Adaptive laboratory evolution (ALE) is a general and effective strategy for optimizing the design of engineered genetic circuits and upgrading metabolic phenotypes. However, the specific characteristics of each microorganism typically ask for exclusive conditions that need to be adjusted to the biological chassis at stake. In this work, we have adopted a do‐it‐yourself (DIY) approach to implement a flexible and automated framework for performing ALE experiments with the environmental bacterium and metabolic engineering platform *Pseudomonas putida*. The setup includes a dual‐chamber semi‐continuous log‐phase bioreactor design combined with an anti‐biofilm layout to manage specific traits of this bacterium in long‐term cultivation experiments. As a way of validation, the prototype was instrumental for selecting fast‐growing variants of a *P. putida* strain engineered to metabolize D‐xylose as sole carbon and energy source after running an automated 42 days protocol of iterative regrowth. Several genomic changes were identified in the evolved population that pinpointed the role of RNA polymerase in controlling overall physiological conditions during metabolism of the new carbon source.

## Introduction

The development of do‐it‐yourself (DIY) technical solutions (Moe‐Behrens *et al*., 2013; de Lorenzo and Schmidt, 2017) for performing adaptive laboratory evolution (ALE) experiments (Portnoy *et al*., 2011; Dragosits and Mattanovich, 2013; LaCroix *et al*., 2017) is expanding the capabilities of researchers to integrate this attractive technique in their regular laboratory workflows. Some examples include the development of automatic microbial cultivation platforms operating mini‐chemostats (Amanullah *et al*., 2010; Bergenholm *et al*., 2019), turbidostats (Marlière *et al*., 2011; Wong *et al*., 2018; McGeachy *et al*., 2019) or segregostats (Sassi *et al*., 2019). Yet, in any circumstance ALE experiments have to be designed taking into account the biological constrains of the evolving microbe and the target to achieve. One of such microorganisms of interest is the soil bacterium *Pseudomonas putida* (in particular strain KT2440) which, because of its distinct management of oxidative stress, has emerged as a prime host of engineered redox reactions (Nikel *et al*., 2016, 2014; Nikel and de Lorenzo, 2018). On this background, we set out to design and implement a DIY framework specifically developed for applying flexible ALE protocols to this bacterium for the sake of increasing its performance as whole‐cell catalyst.

The construction details and every step of the implementation of the evolutionary device are fully disclosed in the Supplementary Information (Materials, Equipment and other procedural features: see Figs S1–S25 and Tables S1–S3). The reader is encouraged to access such accompanying particulars for a more complete comprehension of the technical solution hereby presented. The experimental setup was inspired in the turbidostat scheme proposed by Marlière *et al*. (2011) but was redesigned considering a number of constrains linked to the intrinsic biological features of the KT2440 strain of *P. putida*. One first consideration is that the specimen of interest belongs to a bacterial species that naturally sticks to surfaces and builds considerable amounts of biofilms (Auerbach *et al*., 2000; Espinosa‐Urgel *et al*., 2000; Tolker‐Nielsen *et al*., 2000; Espeso *et al*., 2018). Biofilm formation is operationally problematic, because it clogs culture conduits and selects for surface super‐sticker variants. A second constraint is that *P. putida* KT2440 is strictly aerobic (Nikel and de Lorenzo, 2013; Kampers *et al*., 2019), and proper aeration is required to ensure culture viability and vitality during the long‐term experiment. Furthermore, the evolutionary platform must ensure isolation of the manipulated culture to avoid contamination by microorganisms that may displace the template strain. Finally, growth media quality should be secured at all times for maximizing cell division and foster DNA replication – thereby increasing chances of mutations.

**Fig. 1:**
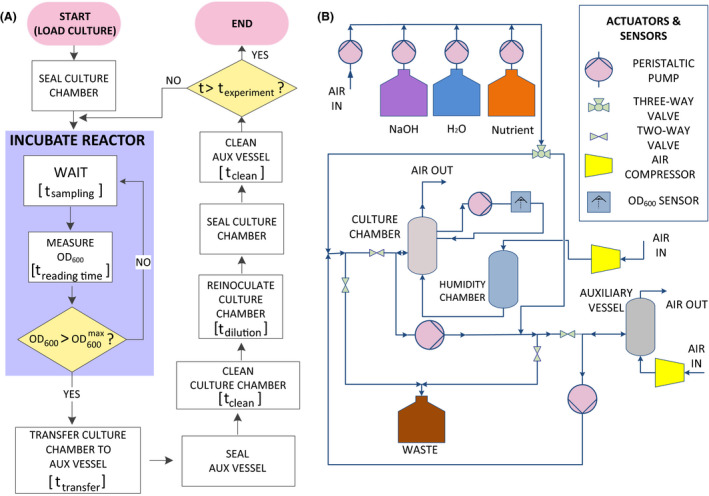
Schematic representation of the DIY device for experimental evolution of *Pseudomonas putida*. A. Block diagram showing the process workflow to implement conceptualized from the ALE protocol taken to analyse as example. The workflow shows the different high‐level actions to perform, their relative order of execution, timing, recurrence loops (i.e. blue square) and decision taking points (on yellow). B. Conceptual scheme showing the actuators, sensors and vessels used to design the fluidic layer of the ALE experimental device. A set of peristaltic pumps, compressors and valves is in charge of transporting different chemicals to clean/wash the vessels and feed a bacterial culture constantly monitored by an optical sensor reader.

With these criteria in mind, an experimental setup was designed and assembled to execute a basic protocol for sustaining bacterial growth for long periods of time. Figure 1A shows the thereby implemented workflow process as a block diagram. The sketch illustrates a recurrent cycle in which a semi‐continuous incubation of a culture is executed by a period of time defined by the user. The protocol included a control loop in the reactor incubation step (blue box) where the workflow was stalled in a periodic subroutine of incubation steps followed by optical density measurements at regular intervals (*t*_sampling_). Such a recurrent sequence ended when an upper threshold OD_600_ value was reached, allowing the workflow in this manner to keep advancing. Figure 1B shows the fluidic layout implemented for succeeding with this protocol. The basic setup includes (i) a bioreactor coupled to a photodetector to obtain OD_600_ readings, (ii) an auxiliary chamber to allow biofilm cleaning with NaOH and H_2_O, (iii) a rack of pumps to deliver the different chemicals and (iv) a group of valves to set the logic of the liquid transport through the circuit. The tubing is connected in a circular fashion with two independent waste outputs and venting connections to ensure an uninterrupted cell culture with sufficient aeration. The design was complemented with electronic and control layers, consistently designed to make possible the synchronized actuation of all these devices (Figs S1–S15). Additionally, this basic arrangement was complemented with the manufacturing of 3D‐printed supports to spatially arrange pumps and valves (Figs S16–S18) and the assembly of an online optical density chamber to gather OD_600_ lectures (Figs. S19–S25).

To test the efficacy of the thereby constructed DIY platform, we used a derivative of *P. putida* KT2440 that had been engineered to grow on D‐xylose, a pentose abundant in hydrolysates of lignocellulosic materials (Chen *et al*., 2017). The construct at stake (named *P. putida* mk‐1, Table S3) bears a large number of genomic modifications for increasing stability, raising the intracellular levels of ATP and NAD(P)H (Martínez‐García *et al*., 2014a, 2014b,2014a, 2014b) and avoiding misrouting of intermediates during d‐xylose metabolism. Specifically, *P. putida* mk‐1 lacks flagella and other energy‐draining cellular devices and has a deletion of *gcd* (thereby lacking glucose dehydrogenase). In addition, the strain bears a chromosomal implant of a synthetic *xylABE* operon encoding XylA (xylose isomerase), XylB (xylulokinase) and XylE (xylose‐proton symporter) from *Escherichia coli* (Dvořák and de Lorenzo, 2018). To this end, the DNA segment bearing *xylABE* was assembled in a mini‐Tn*5* transposon vector (Martínez‐García *et al*., 2014a, 2014b,2014a, 2014b) as described in the Supplementary information. During the construction of the test strain, the mobile element mini‐Tn*5* Sm:: [*P_EM7_* → *xylABE*] was randomly inserted throughout the genome of strain *P. putida* EM42 *∆gcd* (Table S3). The organization of the mini‐Tn*5* transposon was such that the *xylABE* operon could be expressed from the synthetic *P_EM7_* promoter engineered in the mobile element as well as from readthrough transcription of nearby promoters close to the site of insertion.

**Fig. 2:**
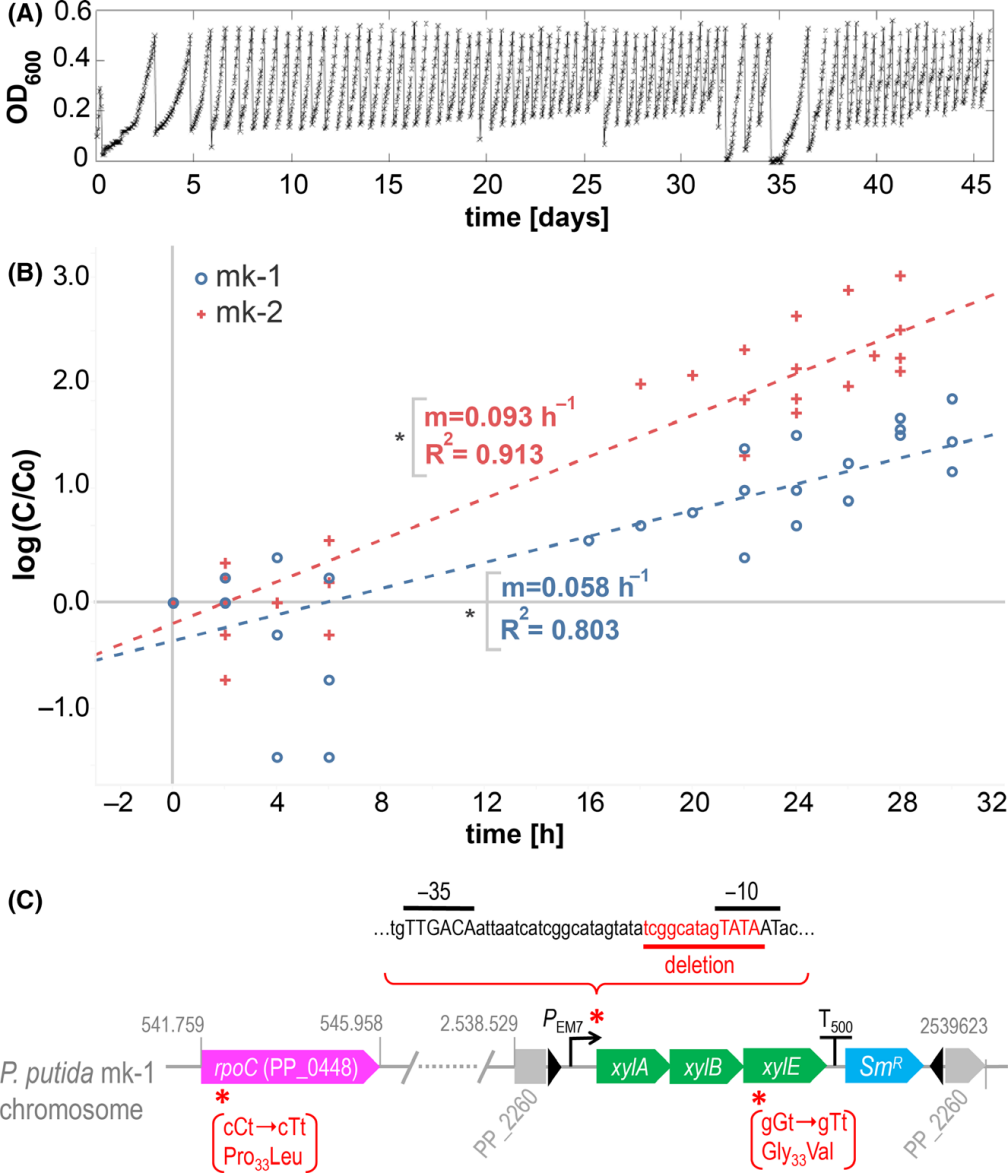
Evolution of an engineered Xyl^+^ strain of *P. putida* along a 42 days protocol of iterative regrowth. A. Optical density evolution during the 45 days period of the ALE experiment. The sawtooth pattern of the graph corresponds to the culture dilution dynamic (semi‐continuous culture) used by the device, programmed to hold the optical density within an exponential growth regime with optical densities within the range [0.1–0.5]. B. Independent ALE validation experiments. Growth curve assays using shake flasks were performed to estimate the growth rates of template (mk‐1, blue) and evolved (mk‐2, red). For the tested conditions, mk‐2 sample exhibited a 60% increment respect to template strain. The plot shows a fitting of three independent biological replicates. Asterisks indicate that both regressions passed *t*‐test at 5% confidence (*P* < 0.05). C. Mutations detected by whole genome sequencing of the *P. putida* mk‐2 sample. A scheme of the *P. putida* mk‐1 chromosome is depicted showing relevant genes and genomic changes detected after the evolution procedure. Genomic coordinates of PP_2260 (locus of mini‐Tn5 insertion) and *rpoC* refer to *P. putida* EM42 ancestral strain. Inverted repeats ME‐I and ME‐O, defining the edges of mini‐Tn5, are also shown by black arrowheads with *xylABE* cluster in between. Locations of detected mutations are denoted by red asterisks. *P*_EM7_ sequence features −35 and −10 boxes in high case and underlined text, while deletion found in mk‐2 genome appears underlined in red colour. Single nucleotide changes found in *rpoC* and *xylE* appear in brackets: wild type and mutated codon are depicted with mutated site in high case. The amino acid change and position in the polypeptide are also shown below.

Selection of the best grower clone on d‐xylose as sole carbon source yielded the aforementioned strain *P. putida* mk‐1 with the business DNA segment inserted in the midst of the locus PP_2260 (a putative glycerol‐phosphate ABC transporter ATP‐binding protein; Fig. 2C). Whether there was a benefit in the interruption of that ORF is unknown, but insertion of the DNA segment with [*P_EM7_* → *xylABE*] in the chromosome secured the stable inheritance of the knocked‐in trait during the course of the bioreactor experiment (Fig. 2A).

Next, strain *P. putida* mk‐1 was inoculated in an intermediate reactor chamber with an operative volume of 20 ml and containing around 10^9^ cultivated cells with an OD_600_ bounded within the range [0.1–0.5]. During a 45 days period, cells were recurrently incubated and diluted (Fig. 2A) using M9 minimal medium supplemented with 0.2% (w/v) d‐xylose and 60 μg ml^−1^ streptomycin. Under these simple conditions, the setup selects for faster growers which – in case of appearance – should bear mutations that increase overall physiological fitness and/or improve nesting of the implanted metabolic segment in the background biochemical network of *P. putida*. The progress of the experiment is shown in Figure 2A. At the end of the corresponding period of time, an increase in growth rate of the population present in the culture became clearly noticeable, same as the fact that beneficial changes occurred probably mainly during the initial phase of the experiment (Fig. 2A, Fig. S26). To examine the basis of such a change, samples were collected from the reactor and further inspected. First, the evolved sample (hereafter called mk‐2) was verified as an authentic descendant of the original strain. For this, the mk‐2 sample was plated on LB and M9 + 0.2% (w/v) citrate agar dishes and cells were streaked out to discard any contamination. Strain clonal identity was confirmed through PCR of the genome with primers 5ʹCTTCAGCTCTTCGCTGTACA3ʹ and 5ʹGCGTGCGCTACAACCTTAC3ʹ that amplify the region surrounding the deletion of the glucose dehydrogenase gene (PP_1444) present in the template strain and which acted as a diagnostic signature. Second, the growth rate of the evolved culture on d‐xylose as the only C source was re‐assessed in respect to the precursor strain performing independent growth curve assays in Erlenmeyer flasks. Regression slopes comparing the two (Fig. 2B) indicated that the evolved specimen grew a 60% faster than template strain. Finally, the genomes of the original *P. putida* mk‐1 strain and the evolved counterpart mk‐2 were sequenced to find mutations that could account for the observed shift in the growth phenotype.

While no modifications became apparent in the bacteria of reference, the faster‐growing derivative bore 3 conspicuous changes in its chromosome. The first modification of the evolved genome was found in the *rpoC* gene of *P. putida*, which encodes the βʹ subunit of RNA polymerase. The *rpoC* of gene of mk‐2 had a point mutation C → T in codon 51 (cCt → cTt) causing a quite drastic change Pro_51_Leu. The emergence of this modification acted in fact as a descriptor of the efficacy of the evolutionary experiment. This is because as a large number of *rpoC* mutations have been reported in the course of laboratory evolution studies aiming to increase *E. coli* growth rate (Cheng *et al*., 2014; Wytock *et al*., 2018; Kavvas *et al*., 2020). Therefore, the Pro_51_Leu change plausibly reflects a similar adaptation in *P. putida*. The other two mutations were identified in the implanted xylose cluster. One of them involved a single base change G → T in the codon 33 of the *xylE* gene (gGt → gTt) which translates into a Gly_33_Val amino acid change. The *xylE* product is a xylose‐proton symporter of *E. coli* composed by several transmembrane domains connected by periplasmic/cytoplasmic amino acid stretches (Davis and Henderson, 1987), and Gly_33_ is located in a periplasmic side close to the H^+^ coupling site Asp_27_ (Madej *et al*., 2014). While a number of loss‐of‐function mutations have been reported for *xylE* (Sun *et al*., 2012), to the best of our knowledge no changes are known to enhance xylose transport. The Gly_33_Val observed in mk‐2 could be one of them, an issue that deserves further studies. Finally, a 13‐bp deletion removing part of the *P*_EM7_ −10 box was observed also in the faster‐growing culture. As mentioned above, *P*_EM7_ is a strong synthetic promoter engineered for driving the expression of *xylABE* cluster in *P. putida* mk‐1 strain. The loss of part of the −10 box (Collado‐Vides *et al*., 1991; Meysman *et al*., 2014) is expected to reduce the promoter activity in the mk‐2 cells. Selective pressure to curb *P*_EM7_ strength might be related to the fact that overproduction of the XylE transporter is toxic to *P. putida* cells (unpublished data). The coexistence in mk‐2 of mutations anticipated to both decrease *xylE* transcription and improve XylE efficiency could reflect a solution to the conflict between the negative effects of overproducing a membrane protein and the need to secure a sufficient inflow of the carbon source for a faster growth. Note that – as discussed above – even complete elimination of the *P*_EM7_ promoter of the genomic implant [*P_EM7_* → *xylABE*] could still deliver expression of the operon owing to readthrough transcription from promoter(s) outside the mini‐Tn*5* insertion (Fig. 2C).

Whether the effects of these three mutations found in the evolved, faster‐growing sample are additive, synergistic or altogether independent is beyond the scope of this technical note and will be the subject of subsequent studies. Correspondingly, further rational engineering cuts and ALE with the constructed system are being considered to remove additional metabolic bottleneck(s) that could have prevented achieving even faster growth of *P. putida* recombinant on the non‐native substrate during the evolution experiment (Elmore *et al*., 2020). Yet, the data presented above accredits the power of the simple and affordable DIY setup described here to generate phenotypes of considerable biotechnological interest in the synthetic biology chassis and metabolic engineering platform *Pseudomonas putida*. Besides the enhancement of catabolic traits, the authors foresee the use of the bespoken device also for the evolution of biosynthetic pathways in *P. putida* and other bacterial cell factories. As additional modules, e.g., for absorbance or fluorescence quantification, can be easily integrated into the presented setup, we entertain the use of this framework also for the accelerated evolution of industrially relevant strains equipped with genetically encoded product‐responsive biosensors (Mahr *et al*., 2015).

## Conflict of interest

The authors declare no competing financial interest.

## Supporting information


**Table S1.** Wiring used in the electric circuit. Table shows the type of connection, which parts of the circuit connects, the type of used wire and the operation voltage.
**Table S2.** Printing parameters used to manufacture the 3D printed supports and equipment.
**Table S3.** Strains and plasmids used in this study.
**Fig. S1.** Device assembly composition scheme. (A) The device is divided in three layers (fluidic layer, electronic layer and control layer) that are also physically separated by design. Every layer is in charge (B) of managing different resources, performing different tasks and receive / deliver different feedback from the other layers.
**Fig. S2.** Schematic illustration of how the different parts of the device are connected by the electronic layer. Fluidic equipment is wired to a switch board, connecting all actuators to a MOSFET Arduino PCB that converts the 5 V control signals delivered by the Arduino Card into effective modulation of 12 V power supply for actuators.
**Fig. S3.** Fluidic hardware control logic acting in each stage of the implemented protocol, and expected transport of liquids within the device.
**Fig. S4.** Fluidic layer assembly blueprint. Every connector, tubing, actuator and sensor participating in the implemented protocol is detailed to make more intuitive how the device was constructed.
**Fig. S5.** ULN2803A MOSFET transistor array scheme and operation regimes. This model contains 8 NPN logic level MOSFET (up) which essentially works as electric gates that regulate the flow of a current passing through them (in Source – Drain direction) by modulating the incoming voltage in the Gate pin, leading to off/on or varying loading regimes (down).
**Fig. S6.** MOSFET Arduino PCB blueprint. Lateral pin holes are placed to match with Arduino pins, allowing an easy fitting. Board size is 100 × 75 mm. Hole Pitch is 2.54 mm. Red and Blue lines depict tracks printed in the front and bottom of the PCB, respectively.
**Fig. S7.** Switch PCB blueprint. Small pin boxes correspond to actuator KK254 switches, which are connected to 16 –pin compacted pin headers host male IDC connectors in charge of linking the board with MOSFET input pins. Real size is 150 × 100 mm. Hole pitch is 2.54 mm. Red and blue lines depict tracks printed in the front and bottom of the PCB, respectively.
**Fig. S8.** Fuse PCB blueprint. Small pin boxes correspond to actuator KK254 switches. Resettable fuse is soldered in the rounded pinholes. PCB size is 35.5 × 12.7 mm. Hole pitch is 2.54 mm. Blue lines depict tracks printed in the bottom of the PCB.
**Fig. S9.** FT232H PCB blueprint. Small pin boxes correspond to actuator KK254 switches. Rounded pinholes are used to solder straight PCB sockets that allow a removable connection with FT232H. D0 and D1 labels define the orientation of the chip. PCB size is 60 × 40 mm. Hole pitch is 2.54 mm. Blue lines depict tracks printed in the bottom of the PCB.
**Fig. S10.** Power board PCB blueprint. Small pin boxes correspond to actuator KK254 switches. Large square labeled as “12V DC IN” is designed to assemble a 4‐way MOLEX mini‐fit switch used to host 12V ATX terminals (typically found in PC power supplies). Additional through holes are available to add a 47 μF and 100 nF condenser to reduce noise coming from the power supply. PCB size is 50 x 20 mm. Hole pitch for KK254 switches is 2.54 mm, and 4.2 mm for MOLEX mini‐fit. Blue lines depict tracks printed in the bottom of the PCB.
**Fig. S11.** Relay board PCB blueprint. Small pin boxes correspond to actuator KK254 switches. Large square labeled is designed to assemble a 12VDC SPDT Relay used to activate or deactivate the air compressor unit, which works at 120/220 VAC. A diode (see arrow) should be used to damp current oscillations when switching ON /OFF the relay. PCB size is 60 × 20 mm. Hole pitch for KK254 switches is 2.54 mm. Blue lines depict tracks printed in the bottom of the PCB.
**Fig. S12.** Images of the assembled device. (A) General overview; (B) Electronic and Control layer; (C) Fluidic layer.
**Fig. S13** Schematic representation of wiring and connector diagram. Each component of the device is connected with a labeled wire. Each wire has a wire type and two terminals that use different connectors.
**Fig. S14.** Example of coding structuring model used to program the Arduino card. Complex high‐level instructions are divided in simpler actions using a cascading decomposition. The codification is then implemented by aggregating these point actions into functions, tested before being used, and then taken as blocks to create more complex routines. This methodology allows minimizing the debugging step and makes easier the code comprehension.
**Fig. S15.** Schematic representation of code structure used to program the Arduino. First an assignation of pins to physical actuators is performed. Next variables used to control the logic of the flow (times to wait, values of OD600, etc.) are included in the code. Then functions in charge of executing operations of increasing complexity to the fluid are defined one by one. Finally, the execution loop calls the highest‐level functions to recurrently apply the required physical actions planned in the cyclic operation to be performed.
**Fig. S16.** Drawing showing for WPX1 pump support design.
**Fig. S17.** Drawing showing WPM pump support design.
**Fig. S18.** Drawing showing valve scaffold design.
**Fig. S19.** Design drawing of the wet chamber.
**Fig. S20.** Images of the optical reader set. Individual parts (down) are screwed with 4 × 25 mm M3 bolts and nuts to assemble a compact closed black box with the wet chamber enclosed in it (up). Only two side holes used to emit 610 nm light and to perform the light measurements.
**Fig. S21.** Design drawing of the chamber scaffold, part I.
**Fig. S22.** Design drawing of the chamber scaffold, part II.
**Fig. S23.** Design drawing of the chamber scaffold, part III.
**Fig. S24.** Calibration slope relating light reading vs optical density in a *P. putida* KT2440 culture grown in M9+0.2% (w/v) glucose using the propose custom‐made optical reader.
**Fig. S25.** OD_600_ readings obtained during the overnight incubation of *P. putida* KT2440 culture grown in M9+0.2% (w/v) glucose using the device.
**Fig. S26.** Growth rate evolution during the experiment development. The sharp peak located at day 30 was a result of a numerical drift caused by the detection of arbitrarily larger values of optical density lectures due to a hardware failure. Once realigned, the optical sensor started working properly. Dashed lines mark the average value ± standard deviation.
**Fig. S27.** Comparison of growth of *P. putida* EM42 Δgcd (control) and mk‐1 strain in rich growth medium and in minimal medium with carbon source. (A) Rich lysogeny broth medium; (B) M9 minimal medium with 5 g l^−1^ glucose. (C) M9 minimal medium with 5 g l^−1^ citrate. Experiment was carried out in microtiter plate (150 μl of medium per well) at 30°C. *P. putida* EM42 Δgcd, filled squares; mk‐1, open squares. Data points shown as means of absorbance A600 of four biological replicates. Standard deviations were within 10% of the mean values.
**Fig. S28.** Stock loading system. By using a loading port (1) and coupling a sterile empty syringe (2), the tubes connecting both bottles are filled with liquid (3), which induce a liquid transfer from the new stock bottle (left) to the empty reactor container bottle (right) by height differences (4) with minimum risk of contamination.
