# Supplementary material for: An automated DIY framework for experimental evolution of Pseudomonas putida
Source: Microb Biotechnol. 2020 Oct 13;14(6):2679–85. doi: 10.1111/1751-7915.13678 (PMC8601172; doi:10.1111/1751-7915.13678)
Supplement: Supplementary file 1 — Table S1. Wiring used in the electric circuit. Table shows the type of connection, which parts of the circuit connects, the type of used wire and the operation voltage. Table S2. Printing parameters used to manufacture the 3D printed supports and equipment. Table S3. Strains and plasmids used in this study. Fig. S1. Device assembly composition scheme. (A) The device is divided in three layers (fluidic layer, electronic layer and control layer) that are also physically separated by design. Every layer is in charge (B) of managing different resources, performing different tasks and receive / deliver different feedback from the other layers. Fig. S2. Schematic illustration of how the different parts of the device are connected by the electronic layer. Fluidic equipment is wired to a switch board, connecting all actuators to a MOSFET Arduino PCB that converts the 5 V control signals delivered by the Arduino Card into effective modulation of 12 V power supply for actuators. Fig. S3. Fluidic hardware control logic acting in each stage of the implemented protocol, and expected transport of liquids within the device. Fig. S4. Fluidic layer assembly blueprint. Every connector, tubing, actuator and sensor participating in the implemented protocol is detailed to make more intuitive how the device was constructed. Fig. S5. ULN2803A MOSFET transistor array scheme and operation regimes. This model contains 8 NPN logic level MOSFET (up) which essentially works as electric gates that regulate the flow of a current passing through them (in Source – Drain direction) by modulating the incoming voltage in the Gate pin, leading to off/on or varying loading regimes (down). Fig. S6. MOSFET Arduino PCB blueprint. Lateral pin holes are placed to match with Arduino pins, allowing an easy fitting. Board size is 100 × 75 mm. Hole Pitch is 2.54 mm. Red and Blue lines depict tracks printed in the front and bottom of the PCB, respectively. Fig. S7. Switch PCB blueprint. Small pin boxes correspond [file MBT2-14-2679-s001.pdf]

Supplementary information to the

BRIEF REPORT

An automated DIY framework for experimental evolution of *Pseudomonas putida*

by

David R. Espeso[1], Pavel Dvořák[2] , Tomás Aparicio[1] and Víctor de Lorenzo[1]*

1. Materials

The following list of materials includes all the components required to assemble the ALE platform used to validate the DiY framework. The material resources, selected standards and metrics for fittings, materials etc. are those used in the presented case study. It is not necessary to strictly stick to used commercial brands: any alternative product satisfying the characteristics of the presented products are valid in the same way. Brands and reference numbers are only provided to be more precise about the specific product purchased by referring it to the manufacturer or distributor.

1.1. Fluidic circuit

- 1 x Silicone tubing, 25 m, 2 mm (id) x 4 mm (od), (VWR, catalog number: 228-0704)
- 1 x Silicone tubing, 25 m, 1 mm (id) x 3 mm (od), (VWR, catalog number: 228-0701)
- 1 x Silicone tubing, 4 m, 4 mm (id) x 6 mm (od), (VWR, catalog number: 228-0709)
- Straight connector, barbed fitting 1/8” ID, polypropylene (Cole-Parmer, catalog number: EW-06365-22)
- Barbed tee connector 1/8” x 1/8” x 1/8”, polypropylene (Cole-Parmer, catalog number: EW-50623-66)
- Barbed tee connector 1/16” x 1/16” x 1/16”, polypropylene (Cole-Parmer, catalog number: EW-06365-77)
- ADCF Male Luer to 1/8” barb adapter, polypropylene (Cole-Parmer, catalog number: EW-30800-24)
- ADCF Female Luer to 1/8” barb adapter, polypropylene (Cole-Parmer, catalog number: EW-30800-08)
- ADCF Male Luer to 1/16” barb adapter, polypropylene (Cole-Parmer, catalog number: SI-45518-00)
- ADCF Female Luer to 1/16” barb adapter, polypropylene (Cole-Parmer, catalog number: SI-45508-00)
- Cole-Parmer Animal Free Male Luer Lock Plug (Cole-Parmer, catalog number: SI-30800-30)
- Cole-Parmer Animal Free Female Luer Fittings, Polypropylene (Cole-Parmer, catalog number: SI-30800-12)
- One-way Luer Check Valves (Female luer- male luer lock) (Cole-Parmer, catalog number: WZ-30505-92)
- 2 x Hosecock clamp (VWR, catalog number: 229-0116)
- 3 x 100 ml laboratory bottles with screw cap DURAN® GL-45 (VWR, catalog number: 215-1514)
- 4 x 1 L Laboratory bottles with screw cap GL-45 (VWR, catalog number: 215-1517)
- 1 x 2 L Laboratory bottles with screw cap GL-45 (VWR, catalog number: 215-1518P)
-1 x 5 L Laboratory bottles with screw cap GL-45 (VWR, catalog number: 215-1519P)
-1 x 5l Corning® Reusable Plastic Low Form Beakers (VWR, catalog number: 1000P-5L)
- 1 x Nalgene™ Square Wide-Mouth Large PPCO Bottle with Closure (Fisher scientific, catalog number: 03-313)
- 3 x Duran 4-Port Assembled HPLC Screw Caps GL-45 (Fisher scientific, catalog number: 10740834)
- Millex Syringe Filter, Fluoropore™ PTFE, Hydrophobic, Non-sterile, 0.45 µm pore size, 13 mm diameter (Sigma-Aldrich, catalog number: SLFHX13NL)
- Cole-Parmer Stopcock with Luer Connections; 1-way; male lock, Non-sterile (Cole-Parmer, catalog number: EW-30600-00)

1.2. Actuators

- Peristaltic Tubing Pump 9-21 ml/min, 12VDC (WELCO, catalog number: WPX1 –P1/16DM4-N)

- Peristaltic Tubing Pump 0.8 – 3 ml, 12VDC (WELCO, catalog number: WPM1 –P1/16BB)
- 2-way isolation valve, barbed, 12VDC (The Lee company, catalog number: LFVA1220110H PEEK)
- 3-way isolation valve, barbed, 12VDC (The Lee company, catalog number: LFRA1220270D PEEK)
- 2x 19 - 37.8L aquarium air pump, 220 AC (Hagen, A842 Aquaclear Air pump 10, catalog number: 15561108447)

1.3. Electronic wiring

- 1x Stranded wire acc to UL, black, style 1007/1569, AWG 18/28 (kabeltronik, catalog number: 0921828)
- 1x Stranded wire acc to UL, red, style 1007/1569, AWG 18/28 (kabeltronik, catalog number: 0921828)
- Molex Female Connector Housing – KK254, 2.54 mm pitch, 3 way, 1 row (RS, catalog number: 296-4940)
- Molex Female Connector Housing – KK254, 2.54 mm pitch, 2 way, 1 row (RS, catalog number: 296-4934)
- Molex Straight PCB header - KK254, 2.54 mm pitch, 3 way, 1 row, through hole (RS, catalog number: 483-8477)
- Molex Straight PCB header - KK254, 2.54 mm pitch, 2 way, 1 row, through hole (RS, catalog number: 483-8461)
- Molex KK 254 2759 Crimp Terminal Contact, Female, 0.05mm² to 0.35mm², 30AWG to 22AWG, Tin Plating (RS, catalog number: 172-9178)
- HARWIN M20, 2.54mm Pitch, 36 Way, 1 Row, Straight Pin Header, Through Hole (RS, catalog number: 547-3150)
- Samtec TSW, 2.54mm Pitch, 10 Way, 1 Row, Straight Pin Header, Through Hole (RS, catalog number: 767-1044)
- Weidmüller PM 5.08, 2 Way 5.08mm Pitch PCB Terminal Strip (RS, catalog number: 425-8720)
- Clever Little Box CLB Series, 2.5 mm Cable Mount DC Jack Plug, 2A (RS, catalog number: 810-4595)
- RS PRO 2.1 mm, 2.5 mm PCB Mount Dual DC Jack Socket, 5A (RS, catalog number: 805-1696)
- ASSMANN WSW AWHW Series 2.54mm Pitch Through Hole IDC Connector, Male, 16 Way, 2 Row (RS, catalog number: 674-1237)
- ASSMANN WSW AWP Series 2.54mm Pitch Right Angle Cable Mount IDC Connector, Socket, 16 Way, 2 Row (RS, catalog number: 674-1101)
- RS PRO 20 Way Unscreened Flat Ribbon Cable, 25.4 mm Width, 10m, (RS, catalog number: 214-0683)
- JST LTO Series Crimp Receptacle, 2.8 x 0.5mm, Tin Plated (RS, catalog number: 166-7517)
- Bematik Faston Terminal, 4.8mm, female (Cablematic, catalog number: FN005)
- Bematik Faston Terminal, 4.8mm, male (Cablematic, catalog number: FN002)
- Bourns 0.5A Hold current, Radial Leaded PCB Mount Resettable Fuse, 60V (RS, catalog number: 647-8493)
- Bourns 2.5A Hold current, Radial Leaded PCB Mount Resettable Fuse, 30V (RS, catalog number: 647-8566)
- Winslow 2.54mm Pitch Vertical 18 Way, Through Hole Turned Pin Open Frame IC Dip Socket, 5A (RS, catalog number: 813-143)
- STMicroelectronics ULN2803A Octal NPN Darlington Pair, 500 mA 50 V HFE:1000, 18-Pin PDIP (RS, catalog number: 714-1167)
- M20-1060200 - HARWIN Female Connector Housing - M20-10, 2.54mm Pitch, 2 Way, 1 Row (RS, catalog number: 681-2812)
- LED lumix, ORANGE, 610 nm, 2500 mcd, 5 mm (RS, catalog number: 888-7164)
- Vishay MRS25 Series Axial Thin Film Fixed Resistor 1.2kΩ ±1% 0.6W ±50ppm/°C (RS, catalog number: 683-3187)

- 1x Socket extension lead (RS, catalog number: 122-1105)
- M20-1060200 - HARWIN Female Connector Housing - M20-10, 2.54mm Pitch, 3 Way, 1 Row (RS, catalog number: 681-2821)
- JST JWPF Male Crimp Terminal Contact 22AWG SWPT-001T-P025 (RS, catalog number: 820-1327)
- RE210-S1, Single Sided Matrix Board with 38 x 61 1mm Holes, 2.54 x 2.54mm Pitch, 160 x 100 x 1.5mm (RS, catalog number: 607-7012)
- HARWIN 2.54mm Pitch 10 Way 1 Row Straight PCB Socket, Through Hole, Solder Termination (RS, catalog number: 681-6826)
- Molex Mini-Fit Plus 46015, 4.2mm Pitch, 4 Way, 2 Row, Straight PCB Header, Through Hole (RS, catalog number: 699-8543)
- SPDT PCB Mount Latching Relay 5 A, 12V dc (RS, catalog number: 718-1761)
- Axial Fixed Resistor 330Ω ±5% 2W (RS, catalog number: 707-8849)
- Vishay 50V 1A, Diode, 2-Pin DO-204AL 1N4001-E3/54 (RS, catalog number: 628-8931)
- RS PRO 47µF 16V dc Aluminium Electrolytic Capacitor (RS, catalog number: 706-0535)
- Tantalum Capacitor 100nF 50V dc Electrolytic Solid ±20% Tolerance (RS, catalog number: 693-3931)

1.4. Automatic control units and computer

- 1x Arduino MEGA 2560 REV3 (RS, catalog number: 715-4084)
- Adafruit FT232H USB TO GPIO/SPI/I2C (Digikey, catalog number: 1528-1449-ND)
- 1x TE Connectivity USB 2.0 Male USB A to Male USB B USB Cable, 1.5m (RS, catalog number: 529-8252)
- 1x Personal computer with LINUX operative system

1.5. Assembly materials and tools

- RS PRO Plier Crimping Tool for Crimp Contact, 24AWG (RS, catalog number: 210-5012)
- Plier set (Amazon, catalog number: Mannesmann - M10808)
- Mini Screwdriver Kit (Amazon, catalog number: Hama 00039694)
- Precision Vernier Caliper (Amazon, catalog number: Mannesmann - M 823-160, 150 mm)
- PU 81 EU - Soldering Station Power Supply, For Weller WSD81 and WSD51 Soldering Station, 450 °C, 80 W, 240 V (Farnell, catalog number: 2629065)
- 0.5mm Wire Lead Free Solder, +217°C Melting Point (RS, catalog number: 682-7564)
- 1 x Stainless Steel, Hex Nut bag, M3 (RS, catalog number: 189-563)
- 1 x Stainless Steel Plain Washer, 0.5 mm Thickness, M3 (RS, catalog number: 189-620)
- 1 x Pan Head Bright Zinc Plated Steel Machine Screw bag, M3, 20 mm (RS, catalog number: 560-625)
- 1x Clear Polyethylene Terephthalate Glycol (PETG) Sheet, 1.2m x 620mm x 1mm (RS, catalog number: 334-6444)
- Dow Corning 6011048 SRS Transparent Silicone Sealant Paste 50 ml Tube (RS, catalog number: 113-008)
- RS PRO 2.85mm White PLA 3D Printer Filament, 1kg (RS, catalog number: 832-0273)

- 1x RS PRO Solvent for use with HIPS Filament (RS, catalog number: 918-1091)
- SMARTFIL 2.85mm Natural PETG Printer Filament, 750 g (Tr3sDland, catalog number: -)
- SMART MATERIALS 2.85mm Support Printer Filament, 750 g (Smartmaterials3d, catalog number: -)
- 1x Electrical Tape, 19 mm x 20m (RS, catalog number: 511-4306)
- Chemtronics 1.5m Desoldering Braid, Width 2.5mm (RS, catalog number: 382-9832)
- RS PRO SC-50 Solder Sucker (RS, catalog number: 771-9543)
- Aluminium foil

1.6. Equipment

- Ultimaker 3, 3D Printer (Tr3sDland, catalog number: -)

1.7. Chemicals

- Dichloromethane 1 l (RS, catalog number: 8222711000)
- Sodium hydroxide, 5 kg (Merck, catalog number: 1064625000)
- D-xylose (Sigma Aldrich, catalog number: W360600-1KG)
- Ethanol 99% (w/v) (VWR, catalog number: 20824.365)
- Sodium hypochlorite (VWR, catalog number: 470302-586)
- $Na_2HPO_4 \cdot 2H_2O$ (Sigma-Aldrich, catalog number: 71643-250G)
- KH_2PO_4 (Sigma-Aldrich, catalog number: P5655-500G)
- NH_4Cl (Sigma-Aldrich, catalog number: A9434-500G)
- NaCl (Sigma-Aldrich, catalog number: S3014-1KG)
- $MgSO_4$ (Sigma-Aldrich, catalog number: M2643-500G)
- Kanamycin sulfate (Sigma-Aldrich, catalog number: K1876-1G)
- Streptomycin sulfate (Sigma-Aldrich, catalog number: S6501-5G)
- 10x M9 salts stock (see Recipes)
- 20% D-xylose (see Recipes)
- 1 M MgSO4 (see Recipes)
- M9 minimal medium supplemented with 0.2% (w/v) D-xylose (see Recipes)

1.8. Recipes

In order to prepare the minimal medium, prepare the following stocks separately:

<u>10x M9 salts stock (for 500 ml of solution)</u>

- 42.5 g $Na_2HPO_4 \cdot 2H_2O$

- 15 g KH_2PO_4

- 2.5 g NaCl

- 5 g NH_4Cl

- 500 ml H_2O

D-xylose 20% (w/v) (or any other desired carbon source, for 100 ml)

- 20 g (w/v) D-xylose

- 100 ml H_2O

1 M $MgSO_4$ (for 100 ml of solution)

- 12.03 g $MgSO_4$

- 100 ml H2O

Filter using a 0.2 µm filter (D-xylose stock) and store all the stocks at room temperature

M9 minimal medium supplemented with 0.2% (w/v) D-xylose (1 L)

100 ml M9 salts 10x

10 ml D-xylose 20% (w/v)

2 ml $MgSO_4$ 1 M

888 ml sterile H_2O

Note: Recipes must be prepared under sterile conditions. Use a 0.45 µm PES filter unit to avoid contaminations and prepare the stocks under demand to prevent contamination and minimize antibiotic degradation.

2. Design Criteria

This framework approaches the implementation of any technical solution (included the one proposed in the main manuscript) into few steps that are sequentially implemented in the following order:

1. Conceptualization of the experiment and workflow design
2. Selection of hardware and assembly
3. Programming of software
4. Device validation: start-up, operation and benchmarking

2.1. Conceptualization of the experiment and workflow design

In this protocol we looked for simplicity, usability, modularity and robustness as main driving principles to draft and organize the presented design. A three-layer scheme (see Supplementary Figure S1A) is proposed to handle wet biology (fluidic layer), power supply / data transfer (electronic layer) and protocol execution (control layer). Every

layer is physically separated from the others because of several reasons: they perform different high-level functions in the device, actuate on the equipment by different ways and receive or send inputs and outputs according to a conceptualized scheme (see Supplementary Figure S1B). Additional reasons such as assembly and maintenance convenience or safety reasons (separation of liquids from the electric circuits to avoid any risk of short-circuit) support this configuration. The most straightforward approach to separate layers is by vertical assembling of the different parts, where fluidic layer would be always located at the bottom of the device and the electronic interface and the control unit at the top of the design.

A

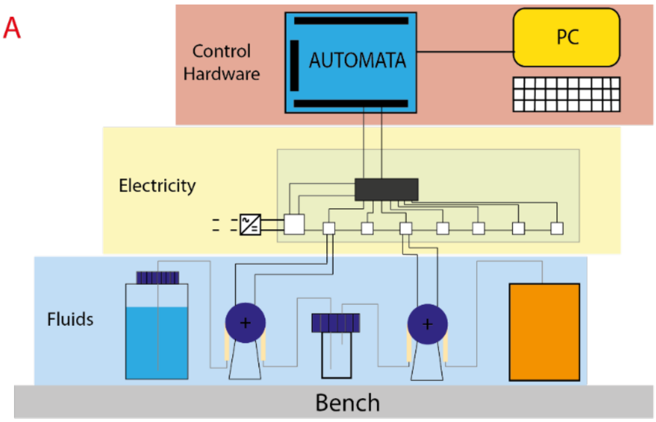

B

	FLUIDIC	ELECTRONIC	CONTROL
**FUNCTION**	Wet experimentation	Power supply<br>Control signal communication	Protocol instruction execution<br>Logic synchronization
**MANIPULATED SUBSTRATE**	Chemicals<br>Microorganism	Electric power (Voltage > 5V)	Electric control signals (Voltage ≤ 5V)
**INPUT / OUTPUT**	Power switching<br>Sensor data<br>Automatic process control	Control signals<br>Sensor data<br>Power switching	User instructions<br>Sensor data<br>Control signals

Supplementary Figure S1: Device assembly composition scheme. (A) The device is divided in three layers (fluidic layer, electronic layer and control layer) that are also physically separated by design. Every layer is in charge (B) of managing different resources, performing different tasks and receive / deliver different feedback from the other layers.

Apart from how to spatially arrange the material resources, the sequence of operations to perform has to be devised. The workflow process contains the set of actions to execute, their sequential ordering and timing (duration and synchronization). It also includes (when applies) any control loop fed with data obtained from the experiment and rules the time constrains not only for the whole process but for each step. Note that special attention must be paid to workflow elaboration, as it will determine in great measure the design of the device lay-out and the programming instructions to implement into the automaton operation routine. The applied iterative workflow steps were following ones:

1- Incubate with a starting OD_{600}^{min} and periodically check OD600 of culture. Repeat this point until a maximum OD_{600}^{max} is reached.

2- Change culture to auxiliary vessel and clean culturing chamber.

3- Clean and wash culturing chamber

4- Dilute culture with fresh nutrient inside the culturing chamber to reach OD_{600}^{min} and discard the rest of the culture.

5- Clean and wash auxiliary vessel

6- If elapsed time has reached the planned duration for the experiment, stop the experiment. Otherwise, restart the loop.

The depicted workflow involves only simple fluid transport operations through a circuit, storage, uptake, dispose of liquids from containers and simple turbidity measurements. The mentioned operations can be successfully performed with basic handling elements (peristaltic pumps, valves, compressors), vessels and simple optic sensors. These data will be used to design the lay-out of the system.

2.1 Layout

If the workflow process details the operation logic of the assembled device, the lay-out presents a physical approach to experimentally materialize the desired protocol. It integrates in a common frame the conceptualization of the protocol to implement with the components required to successfully achieve the planned objectives. It also informs about how the different elements are connected and spatially arranged in shape of assembly blueprints. Assembly schemes were typically categorized by layers attending to which is the physical resource the described hardware will manage. In the presented case it was mass transport (fluidic layer), energy (electronic layer) and information (Control layer). Although segregated, layers are dependent one each other by design. They exchange information of different nature to convert a discretized version of the protocol to implement into an automated manipulation of the different chemicals and biological agents without human supervision. More details about each layer are next included.

2.1.1 Fluidic Layer

The fluidic layer is the part of the framework where the wet experiment takes place. Chemicals and biological agents are transported in fluid phase through a preassembled circuit following a coordinated logic to resemble the protocol to implement. Connection between tubing and components were performed using barbed connectors (described by ASTM D2609 – 15 standard (ASTM, 2015)) and Luer fittings (described by ISO 80369-7:2016 standard (ISO, 2016)). Peristaltic pumps and air compressors were used to pump liquids and air through the circuit, and electromechanical valves helped directing the transport of fluids through the circuit. A battery of pumps was in charge of delivering different chemicals into the circuit. The culture chamber and auxiliary vessel were connected in a circular fashion and had two separated supply and waste lines to permit an independent liquid managing. Two air compressors and a humidity chamber were used to create positive pressure in the vessels (to avoid outer contamination) and bubbling filtered humid air into the culture (which promoted oxygenation and shaking of bacterial suspension). A 3D printed custom-made optical density reader was installed to monitor the cell density of the culture. Finally, a gravity driven refilling system was included in stock bottles to minimize contamination risks during refilling process.

Overall, the fluidic layer manages the experiment itself by performing adding, removing, transporting or monitoring operations in the physical support, which in this case is a fluid medium. It describes all the equipment and complements to make possible such operations. Material resources attending to utility and purpose within the circuit are categorized in four main groups (tubing and fittings, chambers/reactors, stocks, actuators and sensors):

- Tubing and fittings: It includes all the material needed to generate a fluidic circuit in which liquids and gases will be handled (i.e. tubing, connectors, adapters, needles, etc.).
- Chambers: This category comprises all the vessels and containers used to contain the fluids used in the implemented protocol (i.e. stocks of chemicals, reactors, waste recipients, etc.)

- Actuators: Equipment exerting physical effects in the managed fluids during the protocol are included here (pumps, valves, compressors, heating elements, etc.)
- Sensors: Devices able to measure any property of the handled fluids in the device are classified according to this label (light sensor, pressure gauges, etc.)

The equipment has to be carefully selected to avoid unplanned interactions with the handled medium. Followed criteria are included in these bullet points:

- Waterproof / mechanical stability: Materials in contact with the handled fluid must guarantee an impervious behavior combined with a mechanical robustness to maintain the structure of the container when manipulated.
- Chemical compatibility: Handled fluids can carry chemicals of different nature (e.g. polar, organic) that must be chemically compatible in order to prevent the any interaction between the tubing and containers with the handled fluids (swelling, unspecific adsorption/ absorption, corrosion, etc.).
- Biological compatibility: materials of containers hosting the fluid media have to be inert or interact as less as possible with biological agents, except in those cases where it is the case of study.
- Regularity and smoothness: Tubing and containers should be completely regular shape, with an inner design free of dead gaps or corners. Materials of these are strongly recommended to have pore sizes always smaller than the tested microorganism to minimize biomass attachment into the material.
- Minimal contact: Contact surfaces with biological agents should be minimized to avoid contaminations. Avoid the use of mobile parts within fluids carrying microorganisms. Use actuators / sensors that perform their tasks without contacting the handled fluids (i.e. optical sensors, peristaltic pumps, etc.).
- Accessible assemblage: Connections between parts of the fluidic circuit should be easy to set up to boost the construction, modification and checking of the overall layout. The use of threaded fittings is recommended when possible.
- Reusability / low-cost: Performing biological replica involves sterilizing all the parts in the fluidic circuit before each run. Materials are recommended to be autoclavable or resistant to sterilizing agents (for those parts which are expensive) or very cheap to replace them in every new replica.

2.1.2 Control Layer

The control layer is formed by equipment in charge of delivering the instructions to execute in the experiment by means of a programming language. The automaton exerts three functions:

1. It acts as translator between conceptual framework managed by humans in shape of protocols (high level of abstraction, pure information) and the raw procedure-oriented workflow executed by any automated device (low level of abstraction, physical outputs).
2. It provides a physical interface to connect its inner circuitry (responsible of generating the control signals) with the electronic layer, in charge of transporting and converting these signals into effective physical actions
3. It receives any incoming flow of data in shape of electric fluctuations coming from sensors (when apply) and translate it into a digital quantity, which can be used for informative or “online” decision-taking purposes.

In the framework presented, an Arduino MEGA board is used as automaton. Arduino MEGA can be powered by a USB port connection or with a 12V jack port. This unit contains numbered connections (pins) that are connected to

the electronic layer using 2.54 mm pitch straight pin headers. Although all pins can be configured to act as outputs of digital signals, they are hardware limited in some functions. They can be classified into three groups:

- Input pins: Contains an analog to digital converter to process analogic readings coming from sensor. They are typically labeled with the alias Ax (i.e. A0, A1,…).
- Output pins: By using encoded instructions, the voltage of these pins can be modulated to generate electric signals of voltage 0-5V to generate control signals. There are two kinds of output pins in Arduino that limits their use when connected to the different actuators:

    1. Digital pins: These pins (up to 54 pins) can only display an ON / OFF state (0-5V). Any actuator connected to this type of pin is required to operate under this working regime (i.e. a valve).
    2. PWM pins: Arduino does not have any digital to analog converter, thus it is not able to obtain a pure analogic of intermediate voltage (i.e. 3 V). Nevertheless, it is possible to emulate analogic signals within 0-5V range by using pulse width modulation mode (PWM): a train of 0 – 5V pulses is generated at a regulated frequency, whose integral resembles an analogic signal of smaller voltage. This type of pin can operate actuators of varying load depending on the applied voltage (i.e. the rotation speed of a peristaltic pump as a function of the input voltage). There are only 15 PWM pins (marked with a ~ signal), thus the number of actuators requiring this feature is limited and has to be considered as constrain for the design of the framework.

- Communication pins: These pins are used for exchange of data among equipment using serial protocols. Arduino can use different type of serial protocols, which can be grouped in two categories:
    1. Synchronous protocols: The communication between equipment is serialized by using roles. One equip is considered the master of the network, and the rest of the equipment play the role of slaves. The master is in charge of synchronizing the communication over the network with a common clock signal, and identifiers to every slave are assigned. The communication is supervised using start and end signals. When a communication is ongoing, the network finds the target equipment and establishes a data transfer channel between sender and receiver. A failed communication will block the execution of the workflow. This setup may use a shared bus for creating the network of slaves. One example of a synchronous communication would be using I2C protocol using SDA/SCL pins.
    2. Asynchronous protocols: The communication between equipment is not synchronized by a common clock signal but using start and stop signals. There are no assigned master / slave roles. The communication lines are point to point (not a common bus for all connections). A failed communication does not block the execution of the workflow. An example of an asynchronous communication would be connecting 2 UART TX/RX ports or using a FTDI friend USB to serial UART converter (RX/TX)

2.1.3 Electronic layer

It includes all the physical hardware in charge of handling the power supply of equipment acting on the fluidic layer and the traffic of information responsible of synchronizing the activation of the different devices. For security and simplicity, all electronic devices used within this framework were powered using Direct current (DC) at two voltage levels: 12 V for feeding actuators and 5V to operate the control signals generated in Arduino control unit. The Arduino automaton was physically connected to the rest of the circuit by means of PCB shield using 2.54 mm pitch straight pin headers. This shield directly interfaced all the Arduino pins to connect them to transistor arrays, allowing the coupling of 12V and 5V voltage levels. The output of the transistor array (power supply in every electric line) was collected by an IDC connector and transported to a switch panel where the different actuators were connected to the circuit. Sensors were directly connected to Arduino card. A schematic description of the circuit is detailed in Supplementary Figure S2.

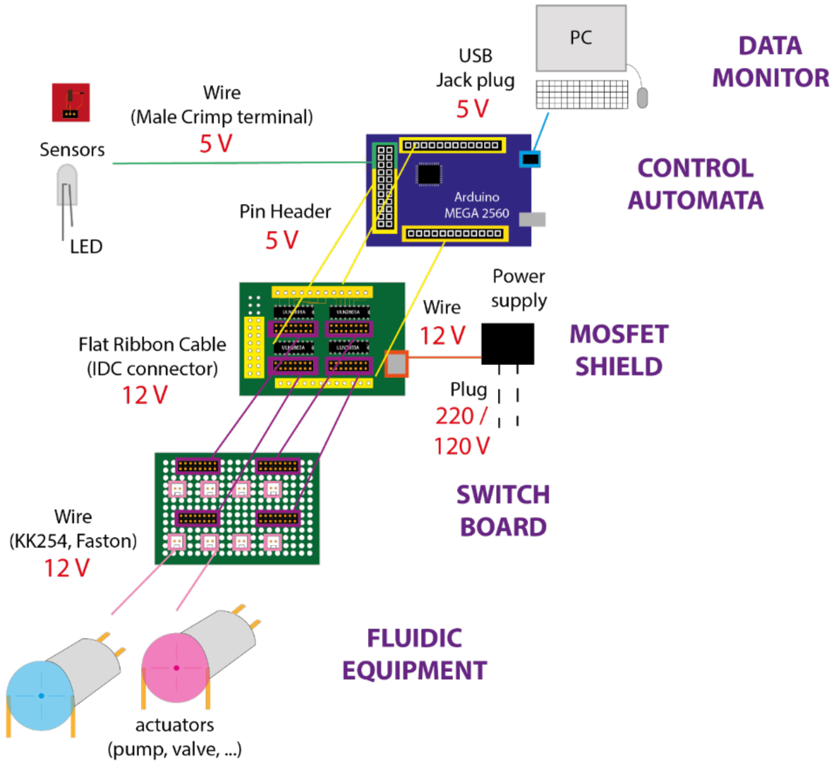

Supplementary Figure S2: Schematic illustration of how the different parts of the device are connected by the electronic layer. Fluidic equipment is wired to a switch board, connecting all actuators to a MOSFET Arduino PCB that converts the 5V control signals delivered by the Arduino Card into effective modulation of 12V power supply for actuators.

More specifically, there are several types of components required to do this:

- Printed Circuit Boards (PCBs): PCBs are silicon made supports that can be customized to connect different electronic components using conductive tracks within the same physical substrate in a compact design.
- Switches and connectors: Equipment use different kind of connectors according to the manufacturer and the type of device. Switches allow plugging them in into the electric circuit by means of a stable but removable connection.
- Electric wiring: When the physical distance among components is large, wires are used to link connect them into the designed circuit. It may be used in shape of regular wires. Wires must be chosen / designed to support the intensity of the current passing through them.
- Transistor arrays: The central component of the electronic layer used in this framework is the transistor array: an electronic component packing eight metal–oxide–semiconductor field-effect transistors (MOSFET). Each individual MOSFET acts as a current regulator: any electric line with a certain voltage and intensity entering inside these components (i.e. the power supply of an actuator) can be modulated by using an auxiliary current much weaker in intensity and voltage (a control signal provided by an automata). The MOSFET current modulation allows resembling not only switching on/off dynamics but also an effective analogic voltage regulation of the controlled current, enabling the control of analogic actuators at their full operation range. As a consequence, low power control signals (carrying the logic of the implemented protocol) are converted into an effective device operation by managing the actuator power supply dynamics.
- Fuses and auxiliary lights: Electric equipment should always contain security measurements to avoid risk of fire due to unexpected short circuits. Fuses are typically attached as a part of the circuit to shut down the electric current if this exceeds a secure limit. Fuses can be resettable or work as a single use circuit breaker. Auxiliary lights are supporting elements (thus not necessary) to visually control the proper operation of the electric flow through the circuit.

- Power supply: The power supply feeds the assembled device with electric power to actuate all the equipment.

Electric network operates at 120 / 220 V AC, but millifluidic actuators typically works at much lower voltages and direct current. A current transformer is then required. Regular PC power sources transform AC current from the electric network directly into a range of power lines operating at different voltages, depending on the specification of the manufacturer. Typical used values are 12 and 24 V.

2.2. Hardware actuation logic

The workflow presented in the main manuscript can be schematically reorganized into five high-level protocol stages, in which the different actuators switch ON and switch OFF to execute the protocol of interest (Incubation, culture transfer, cleaning culture chamber, reinoculation and cleaning of auxiliary vessel). Supplementary Figure S3 shows an illustration showing which equipment participate in each step and the direction of the flow moving through the device.

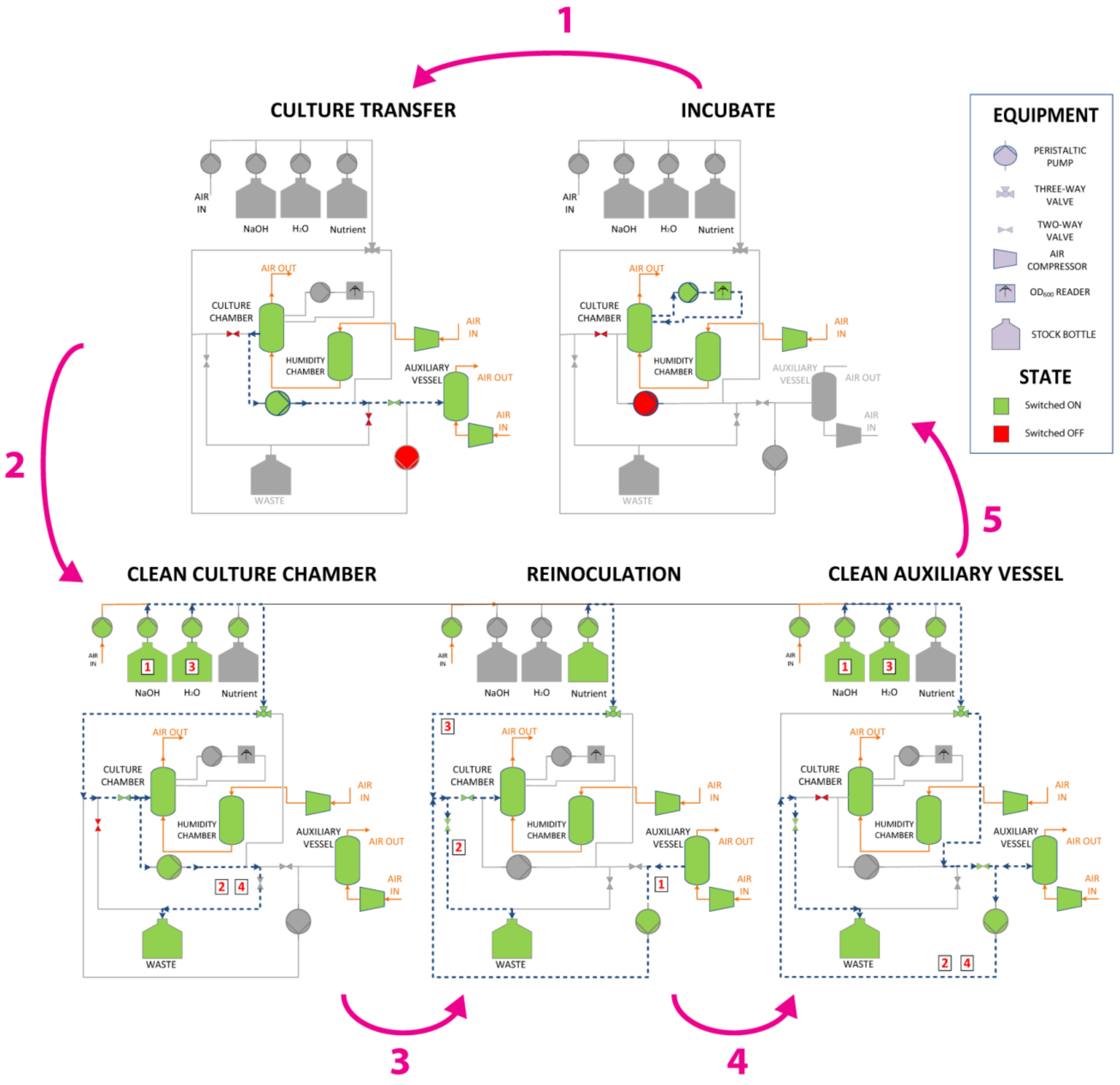

Supplementary Figure S3: Fluidic hardware control logic acting in each stage of the implemented protocol, and expected transport of liquids within the device.

3. Assembly

Once the design was performed and all the required materials have been purchased, the next step was to assemble the device. The device was first assembled by layers, from bottom to top as illustrated in Supplementary Figure S1A: first the fluidic, then the electronic and finally the control unit and the programming of the automata. The performed actions are described next:

3.1. Fluidic layer

1- The execution of protocols in which fluid transport is a must demands the use of electrically powered actuators and tubing (pumps, valves, tubes). All the equipment fed with electricity must be safely placed out of potential areas where liquid may be spilt onto them. Hence the actuators were installed onto a custom-made scaffold to fulfill this security measure. The set of CAD planes to manufacture supports for them using a 3D Printer is available upon request and/or can be downloaded from https://www.dropbox.com/sh/gt1m0e0i8wd1wfh/AAC5Ag5Z7IT7XMcp7-zrS1Cwa?dl=0 (see below). Stackable printed supports help separating wet area of the fluid layer from the electric wiring and connectors required to power actuators and control. However, any other alternative allowing such physical separation can be valid.

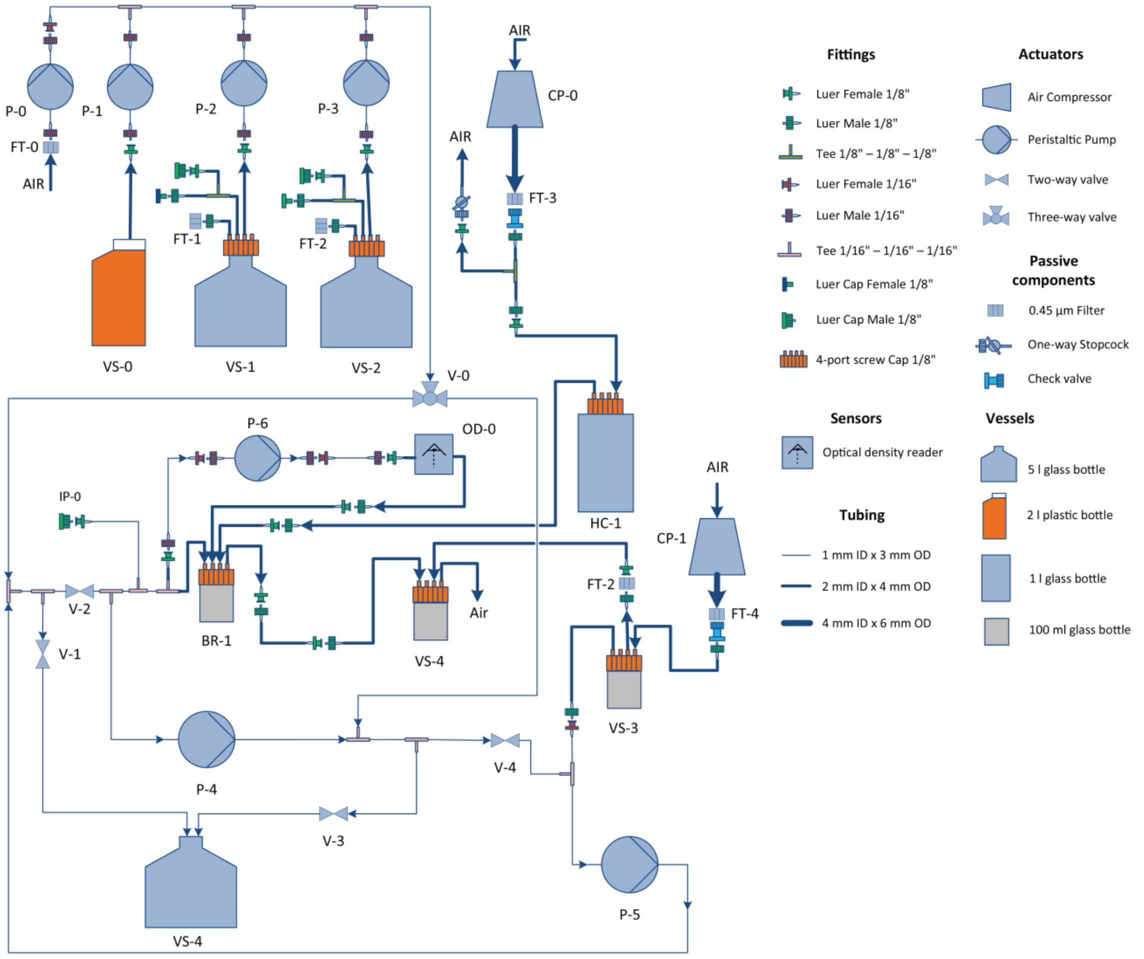

Supplementary Figure S4: Fluidic layer assembly blueprint. Every connector, tubing, actuator and sensor participating in the implemented protocol is detailed to make more intuitive how the device was constructed.

2- Place the stock bottles close to the pumps in charge of pumping the liquids into the device. Also place the vessels which will contain the culture in front of the equipment rack, the compressors, the humidity chamber and the waste container. Allocating all these will allow you to know what is the required length of the tubing to join all the equipment and vessels.

3- Manufacture and assemble the OD_{600} chamber using a 3D printer as described section S8. Place the chamber in vertical position to avoid the presence of bubbles within the measurement cell.

4- Assemble the fluidic circuit using the fittings and passive elements as depicted in the assembly scheme (Supplementary Figure S4). It illustrates the assembly blueprint followed to assembly the fluidic layer. It is decomposed in its physical components (fittings, tubing, actuators, etc.) to guide the reader about how the device was assembled. Sample injection and removal are performed using tube IP-0 with a sterile syringe prepared to fit with Luer connections.

3.2. Electronic layer

The following steps to assemble this layer were the following:

5- Prepare required wiring to connect all the electronic components and assemble components in PCBs following the scheme provided below (PCB Gerber manufacturing files are available upon request and/or can be downloaded from https://www.dropbox.com/sh/gt1m0e0i8wd1wfh/AAC5Ag5Z7IT7XMcp7-zrS1Cwa?dl=0)

6- Locate the PCBs out of the reach of any liquid. Add fuses to all equipment (fuse board) and place them onto the printed supports, and use a two-level tray or a support to physically separate tubing (lower part) from circuits (upper part). Place the Power board, FT232H board, relay board, switch board and Arduino card in the upper part of the tray.

7- Fit MOSFET - Arduino board to Arduino card.

8- Place the power supply in a high position out of reach of any liquid.

9- Wire all PCBs following the provided scheme.

10- Connect the power supply to the electric network

Here, PCBs blueprints used to assemble the electronic layer are included ready to be used. Also, it is detailed how to wire them properly. The presented manuscript also includes the manufacturing files (Gerber files). The PCBs contains the holes to host all the components and the tracks that join them. Individual components have to be assembled manually by soldering. Alternatively, PCBs can be also made by using drilled boards and manually creating the tracks using regular soldering.

3.2.1. MOSFET – Arduino Shield

This PCB directly interfaces Arduino card to physically connect Arduino pins operating at 5V with 12 V layer acting the fluidic layer actuators. The key element in the layer is ULN2803A transistor array, which acts as an electronic valve able to regulate the power flowing through them (Supplementary Figure S5). Every array contains 8 individual MOSFET, with a maximum allowable intensity of 500 mA.

A blueprint of the MOSFET – Arduino PCB shield is presented (see Supplementary Figure S6). It is a simplified version of a PCB model kindly shared by L. Cronin (Yoshida, et al., 2017). To assemble the board, simply follow these steps:

1- Solder Straight Pin Header in all holes of the plate except the ones labeled as POWER and ULN2803A. Solder 18 (TX1), 19 (RX1) communication pins using special Samtec TSW Straight Pin Header (they have double length to connect RX / TX cable) to Arduino card. Be sure that pins are soldered in straight position and not partially inclined.

2- Solder 4 open Frame IC Dip Socket (18 pin) on the ULN2803A label

3- Solder a 5.08mm Pitch PCB Terminal Strip in the POWER labeled pins

4- Mount ULN2803A transistor arrays onto IC Dip Sockets by carefully pressing the chip onto the Socket without bending the IC legs

5- Connect the shield with the Arduino card by matching the lateral pin arrays, being sure that all of them are inside the Arduino pins

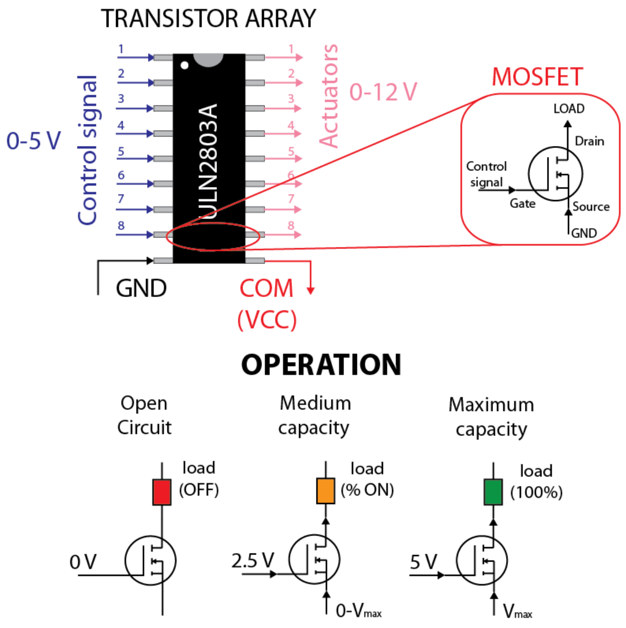

Supplementary Figure S5: ULN2803A MOSFET transistor array scheme and operation regimes. This model contains 8 NPN logic level MOSFET (up) which essentially works as electric gates that regulate the flow of a current passing through them (in Source - Drain direction) by modulating the incoming voltage in the Gate pin, leading to off / on or varying loading regimes (down).

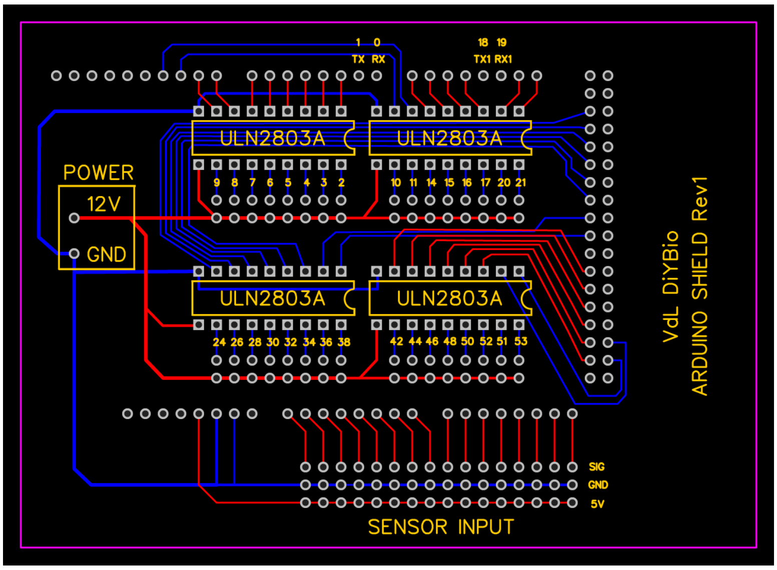

Supplementary Figure S6: MOSFET Arduino PCB blueprint. Lateral pin holes are placed to match with Arduino pins, allowing an easy fitting. Board size is 100 x 75 mm. Hole Pitch is 2.54 mm. Red and Blue lines depict tracks printed in the front and bottom of the PCB, respectively.

Note: Sensor input pins (see pins at the lower part of the card) can be actually modified to work either as 5V analog input or digital outputs. Hence these pins can be used to switch ON/OFF 5V operated elements (i.e. LEDs) by connecting Signal and Ground terminals as positive and negative terminals.

3.2.2. Switch board

The switch board (Supplementary Figure S7) is an auxiliary PCB used to reduce the length of the wiring connecting the actuators with the MOSFET-Arduino Shield. It collects all the equipment switches and channels their signal using a Ribbon flat cable headed with an IDC type connector. The other side of the IDC connector is switched directly on the MOSFET interface. Supplementary Figure S5 shows the blueprint of the board.

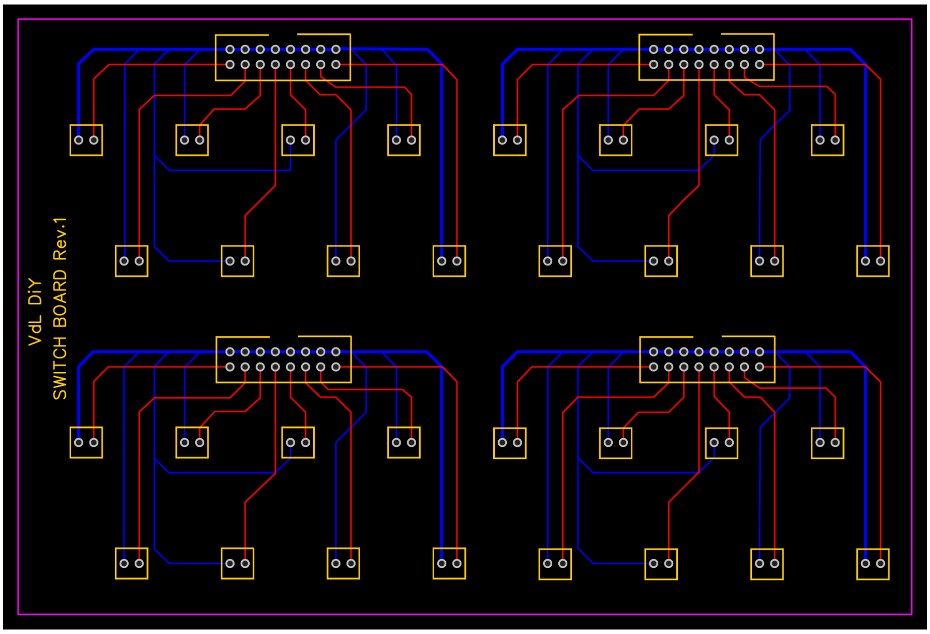

Supplementary Figure S7: Switch PCB blueprint. Small pin boxes correspond to actuator KK254 switches, which are connected to 16 –pin compacted pin headers host male IDC connectors in charge of linking the board with MOSFET input pins. Real size is 150 x 100 mm. Hole pitch is 2.54 mm. Red and blue lines depict tracks printed in the front and bottom of the PCB, respectively.

To assemble the board, simply follow these steps:

1- Solder 2-way male KK254 straight PCB headers in the pin holes labeled with a small square.

2- Solder 16 Way- two row solder Pitch-through hole IDC connectors in the pin holes labeled with a long rectangle.

3.2.3. Fuse Board

The fuse board is a small PCB which interfaces the actuator with the switch panel, introducing a resettable fuse for every actuator. This will avoid risk of fire due to short circuits due to equipment malfunctioning. PCB blueprint is included next (see Supplementary Figure S8):

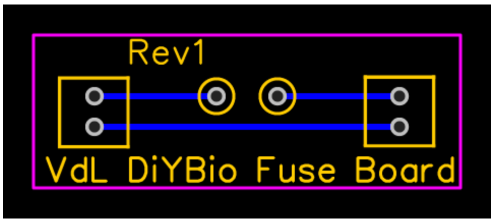

Supplementary Figure S8: Fuse PCB blueprint. Small pin boxes correspond to actuator KK254 switches. Resettable fuse is soldered in the rounded pinholes. PCB size is 35.5 x 12.7 mm. Hole pitch is 2.54 mm. Blue lines depict tracks printed in the bottom of the PCB.

To assemble the board, simply follow these steps:

1- Solder 2-way male KK254 straight PCB headers in the pin holes labeled with a small square.

2- Solder a Radial Leaded PCB Mount Resettable Fuse in the middle part of the plate (use the two pins with a circular label).

3.2.4. FT232H board

The FT232H board is a small PCB that interfaces the Adafruit FT232H Breakout with a connector to fit a wire directly connecting UART pins D0 and D1 in this chipset with Arduino TX1 / RX1 communication port. Blueprints are presented in Supplementary Figure S9:

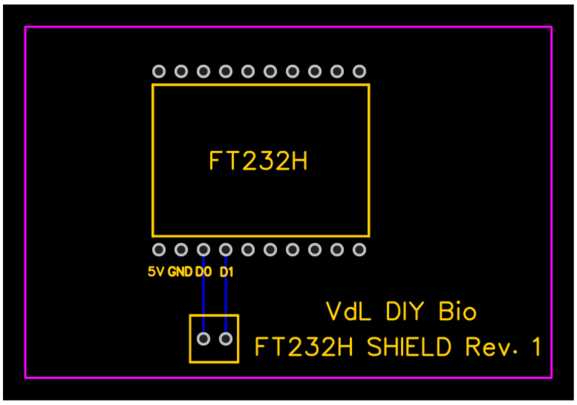

Supplementary Figure S9: FT232H PCB blueprint. Small pin boxes correspond to actuator KK254 switches. Rounded pinholes are used to solder straight PCB sockets that allow a removable connection with FT232H. D0 and D1 labels define the orientation of the chip. PCB size is 60 x 40 mm. Hole pitch is 2.54 mm. Blue lines depict tracks printed in the bottom of the PCB.

To assemble the board, simply follow these steps:

1- Solder 2-way male KK254 straight PCB headers in the pin holes labeled with a small square.

2- Solder 10-way Row Straight PCB Socket in the pin holes labeled with a the FT232H label

3- Mount FT232H chipset onto straight PCB Sockets by carefully pressing the chip against the board without bending the IC legs. Select the proper chip orientation in such a way D0 and D1 pins are coincident with PCB labels.

3.2.5. Power Board

This board (blueprint in Supplementary Figure S10) acts as a switch panel to supply 12V current to the different devices in which they are required (Arduino Jack Port, MOSFET-Arduino board).

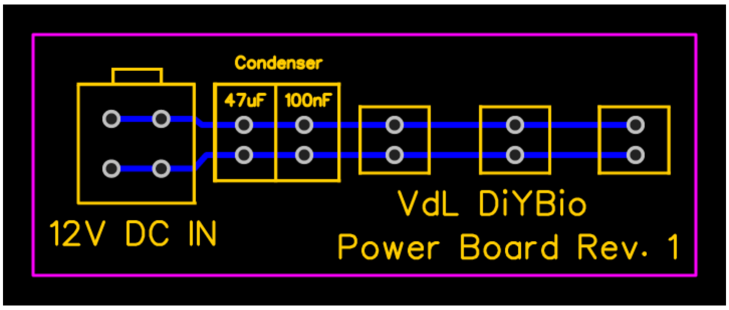

Supplementary Figure S10: Power board PCB blueprint. Small pin boxes correspond to actuator KK254 switches. Large square labeled as “12V DC IN” is designed to assemble a 4-way MOLEX mini-fit switch used to host 12V ATX terminals (typically found in PC power supplies). Additional through holes are available to add a 47 µF and 100 nF condenser to reduce noise coming from the power supply. PCB size is 50 x 20 mm. Hole pitch for KK254 switches is 2.54 mm, and 4.2 mm for MOLEX mini-fit. Blue lines depict tracks printed in the bottom of the PCB.

To assemble the board, simply follow these steps:

1- Solder 2-way male KK254 straight PCB headers in the pin holes labeled with a small square.

2- Solder 4-way Molex Mini-Fit Plus in the 4 pinholes labeled as 12V DC IN

3- Solder 47 *µF and 100 nF* condenser to close the circuit

3.2.6. Relay Board

This PCB is thought to operate a 12VDC Relay in charge of allowing the connection / disconnection of a commercial air compressor, which work at 120 / 220V AC. Supplementary Figure S11 show the respective blueprint.

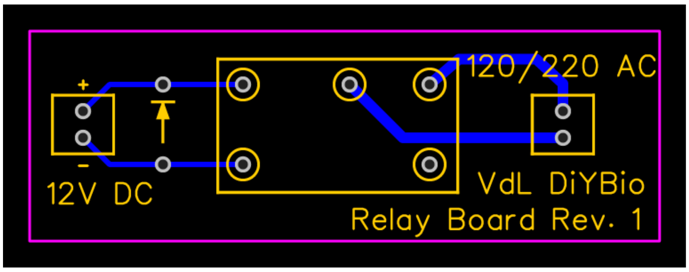

Supplementary Figure S11: Relay board PCB blueprint. Small pin boxes correspond to actuator KK254 switches. Large square labeled is designed to assemble a 12VDC SPDT Relay used to activate or deactivate the air compressor unit, which works at 120 / 220 VAC. A diode (see arrow) should be used to damp current oscillations when switching ON /OFF the relay. PCB size is 60 x 20 mm. Hole pitch for KK254 switches is 2.54 mm. Blue lines depict tracks printed in the bottom of the PCB.

To assemble the board, simply follow these steps:

1- Solder 2-way male KK254 straight PCB headers in the pin holes labeled with a small square. Be sure to solder the header so positive terminal of the wire match with labeled positive terminal.

2- Solder SPDT Relay in the pinholes marked with yellow lines.

3- Solder 1N4001 diode. Special attention must be paid to polarity when soldering.

3.3. Control layer

Steps to prepare control layer were the following:

11- Connect the light sensor and the LED directly onto the MOSFET Arduino board by using the Sensor input pins. Remember to configure their mode when programming the Arduino Card.

12- Connect the RX / TX port of Arduino board to the TX / RX entrance of the FT232H board.

Supplementary Figure S12 show some images of the assembled device used to perform the reported experiments in the main manuscript is illustrated. Note that, depending on the choice of materials, the outlook of the device may change:

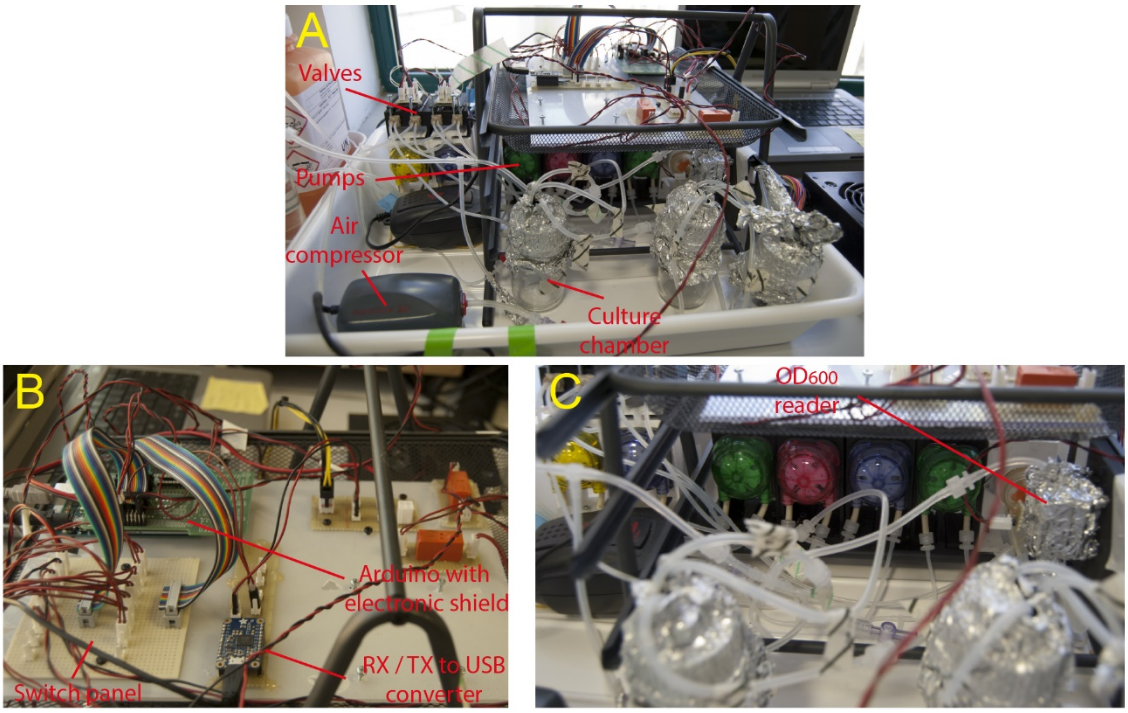

Supplementary Figure S12: Images of the assembled device. (A) General overview; (B) Electronic and Control layer; (C) Fluidic layer

4. Wiring

Wiring is required to transport electric current through the whole circuit. As different connectors use different terminals, cables have to be customized to fit into their respective connectors. A scheme (see Supplementary Figure S13) showing which wires are required, whose are their terminals and which type of wire is used is provided to make more intuitive the assembly. Note that all cables use two filaments (positive and negative). Terminals of the wires according with the used connector: crimped, soldered, screwed or coupled. For more information about how connect each type of terminal to crude wires, use any internet multimedia platform to find a tutorial.

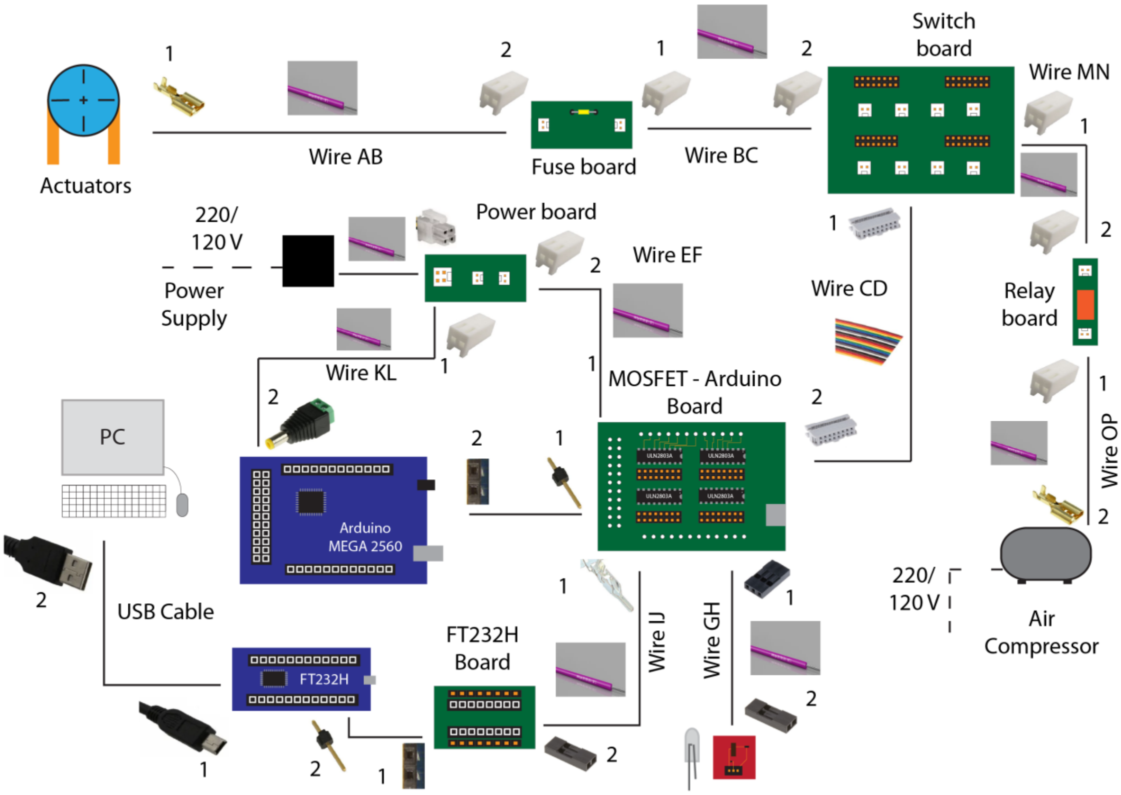

Supplementary Figure S13: Schematic representation of wiring and connector diagram. Each component of the device is connected with a labeled wire. Each wire has a wire type and two terminals that use different connectors.

A brief summary is included in Supplementary Table S1. Numbers and names of wires correspond to labels used in the previous Supplementary Figure.

Supplementary Table S1: Wiring used in the electric circuit. Table shows the type of connection, which parts of the circuit connects, the type of used wire and the operation voltage.

WIRE NAME	Connectors		Circuit connections		Voltage	Wire
	Terminal 1	Terminal 2	Terminal 1	Terminal 2		
**AB**	Female Faston terminal, 4.8 mm	Molex KK254 female housing, 2 way, 1 row	Actuators	Fuse board	12 V	AWG 18/28 wire
**BC**	Molex KK254 female housing, 2 way, 1 row	Molex KK254 female housing, 2 way, 1 row	Fuse Board	Switch Board	12 V	AWG 18/28 wire
**CD**	IDC connector, 16 way, 2 rows	IDC connector, 16 way, 2 rows	Switch Board	MOSFET board	12 V	Flat Ribbon cable
**EF**	Nude	Molex KK254 female housing, 2 way, 1 row	MOSFET Board	Power Supply	12 V	AWG 18/28 wire
**GH**	Harwing Female connector, 3 Way	Harwin Female connector, 3 Way, 1 row	MOSFET Board	Sensors / LEDS (5V equipment)	5V	AWG 18/28 wire

**IJ**	JST Male Crimp Terminal 22AWG	Harwin Female connector, 2 Way, 1 row	MOSFET Board	FT232H	5V	AWG 18/28 wire
**KL**	Molex KK254 female housing, 2 way, 1 row	Jack Clever 2.5 mm	Power Board	Arduino	12V	AWG 18/28 wire
**MN**	Molex KK254 female housing, 2 way, 1 row	Molex KK254 female housing, 2 way, 1 row	Switch Board	Relay Board	12V	AWG 18/28 wire
**OP**	Molex KK254 female housing, 2 way, 1 row	Female Faston terminal, 4.8 mm	Relay Board	Compressor wire	220V	AWG 18/28 wire
**USB Cable**	MiniUSB	USB	FT232H	PC	5V	Cable

5. Programming

Programming the Arduino card to execute the protocol of interest is as important as the physical assembly of the device. It involves dispatching to the automaton the set of instructions required to obtain a consistent sequence of actions in the manipulated biological agent through the use of the different actuators included in the fluidic layer. The Arduino code for the semi-continuous log-phase bioreactor is available upon request and/or can be downloaded from https://www.dropbox.com/sh/gt1m0e0i8wd1wfh/AAC5Ag5Z7IT7XMcp7-zrS1Cwa?dl=0.

Supplementary Figure S14: Example of coding structuring model used to program the Arduino card. Complex high-level instructions are divided in simpler actions using a cascading decomposition. The codification is then implemented by aggregating these point actions into functions, tested before being used, and then taken as blocks to create more complex routines. This methodology allows minimizing the debugging step and makes easier the code comprehension.

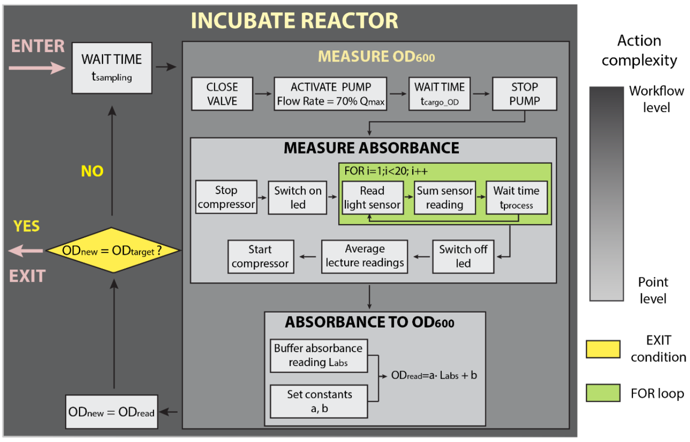

There are many valid programming implementations for a specific workflow. However, some of them are more advantageous than others in terms of design, resilience and code debugging. In our technical proposal we used a structured function based protocol of hierarchical complexity: it consisted on defining simple functions implementing simple protocol operations (i.e. wait time, receive liquid in reactor X, discharge reactor A to waste, etc.) that were then used as basic functions to implement more complex routines (i.e. dilute culture, transfer culture, etc.) until covering all the steps planned in the process workflow. The use of a stratified set of functions ordered by relative complexity allowed a very simple code debugging and implementation: when lowest complexity functions are individually validated first, the upper level functions containing them are much less eager to show an improper functioning. The programming bugs are thus bounded to a much small set of code lines (the upper level functions), that once they are fixed, the validation process continues until the whole workflow action is fully covered. Supplementary Figure S14 shows an example of how point instructions and function-based instructions can be combined forming a sequential cascading of

commands leading to execute a high-level instruction typically included in the process workflow. Additionally, Arduino programs to test, setting up and operate the device, together with a Python program to control the Arduino-PC communication are available upon request and/or can be downloaded from https://www.dropbox.com/sh/gt1m0e0i8wd1wfh/AAC5Ag5Z7IT7XMcp7-zrS1Cwa?dl=0

5.1. Arduino code (available upon request and/or can be downloaded from https://www.dropbox.com/sh/gt1m0e0i8wd1wfh/AAC5Ag5Z7IT7XMcp7-zrS1Cwa?dl=0)

Several key concepts must be taken into account when conceptualizing this step:

1. The protocol to implement has to be first decomposed in a sequence of chronologically ordered discrete actions.
2. There are two types of actions according to the level of complexity: point actions translate into single isolated actuations in fluidic devices (i.e. open valve, switch on light, etc.) actuated by switching the voltage state of an Arduino pin. Protocol actions involve a combination of several point actions to generate a higher-level effect (i.e. transfer culture from vessel A to B would need activating pump 1, open valve 2 and close valve 3).
3. Point actions are virtually instantaneous (require milliseconds to be executed, i.e. open a valve), whereas protocol actions require a user defined time that ensures the action is successfully performed (i.e. wail the required time to transfer all the liquid from A to B).
4. Protocol actions can be composed by other protocol actions to increase the complexity of the automata actuation (i.e. incubating a culture may involve checking the OD_{600} every hour, which involves pumping a liquid from a chamber A to a chamber B, halt the pumping, activating a led and a sensor, and pump the liquid to the waste container).
5. The Arduino card cannot perform two actions at the same time.
6. Idling in Arduino is an action *per se*, thus no additional actions can be performed during waiting periods unless you define an alternative way of spending time doing something in the meantime (i.e. you can order Arduino check a sensor measurement every 2 minutes during a pause period of arbitrary length).
7. Arduino works by executing any set of instructions in an endless loop. This is ideal for protocols recurrently executed over large periods of time, but a finish condition must be included in the set of instructions to halt the protocol execution when required.
8. Standard logic constructions can be implemented in Arduino programming language. IF conditioning allow comparing a variable of interest and perform different actions according to its value, and FOR / WHILE looping are used respectively to iterate a sequence of actions for a fixed number of times or until reaching an exit condition.

The Arduino program used to perform the experiment is provided with the rest of supplementary material. The content is structured described in Supplementary Figure S15. Additionally, a sterilizing program and an electric test protocol are included to setting-up the device prior to start the experiment. Nevertheless, such code is just an example of how to program a protocol, not the best nor the most efficient. The readers are strongly encouraged to modify and propose modifications to adapt it to their own purposes, even scratching everything and starting from zero.

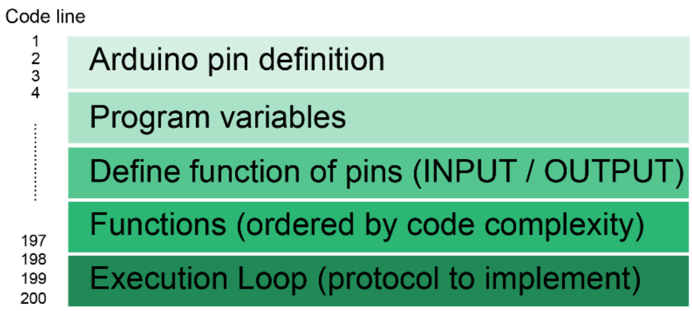

Supplementary Figure S15: Schematic representation of code structure used to program the Arduino. First an assignation of pins to physical actuators is performed. Next variables used to control the logic of the flow (times to wait, values of OD_{600}, etc.) are included in the code. Then functions in charge of executing operations of increasing complexity to the fluid are defined one by one. Finally, the execution loop calls the highest-level functions to recurrently apply the required physical actions planned in the cyclic operation to be performed.

5.2. Python code for PC (available upon request and/or can be downloaded from https://www.dropbox.com/sh/gt1m0e0i8wd1wfh/AAC5Ag5Z7IT7XMcp7-zrS1Cwa?dl=0)

In the proposed assembly, a Python program is executed in a Linux terminal. It is in charge of receiving the sent values by the Arduino card and storing the OD_{600} lectures values in a *.csv file for further analysis. It also displays the whole series of OD_{600} as a raw list in the console.

6. Communication port configuration

Communicating PC and Arduino using USB can be cumbersome because USB ports use to be configured by default to stablish periodic communications, and not to have a communication constantly opened and ready to receive data. Furthermore, when periodic connections happen, or other USB ports are used, the operative system may change the name of the device of interest. As a consequence, such device has not a unique assigned name, but several pseudonames arbitrarily changing. Because ALE experiments have duration of several days, or weeks, a special operative system rule to control the used USB port as a 24h open communication channel is required. Here it is detailed how to achieve when working using Linux Ubuntu 16.04.

1- First it is recommended to install the following packages / software. Open a terminal and use the package provider tool (PIP)

- Python 2.7
- Pyserial
- NumPy / SciPy
- IDE Arduino

2- Next log as superuser with the Adafruit FT232H connected to the PC.

>> sudo su

>> Enter password here

3- Find the USB name assigned to your device

>> ls /dev/ttyUSB* or >> ls /dev/ttuACM*

4- Obtain the device configuration parameters

>> udevadm info -a -p /sys/bus/usb-serial/devices/ttyUSB0

Remember the USB port at which you connect it

5- Create the rule using a singular attribute of the connected device and one of the associated master devices. To do that, first open the rule file:

>> sudo nano /etc/udev/rules.d/98-usb-serial.rules

Then create the rule. For example:

KERNELS=="1-3.3:1.0", DRIVERS=="ftdi_sio", MODE="0666", SYMLINK+="The selected name of your device"

6- Check that your rule is well written. Type:

>> udevadm test /sys/bus/usb/devices

If your rule is not correct, a warning message should appear after the test

7- Now load the rule by typing:

>>udevadm control --reload-rules

8 – Restart the computer and check that the rule is properly working. Open a terminal and type:

>>ls /dev/

You should see the chosen name of your device in the list

<u>Warnings:</u>

- Connect your device ALWAYS to the same USB port used to apply the previous rule, as it links a name to a physical route.
- Check that common terminals (ground) in both PC and Arduino are shared. If they are not, the communication could not work properly.

Finally, once you have uploaded your Arduino Program to the card using the IDE interface, modify USB behavior to avoid periodic resetting when ports are refreshed. To do that, open a terminal, log in as superuser and write the following command

>> stty –F /dev/"your device name" -clocal –hupcl

>> stty –F /dev/ACM0 -clocal –hupcl (If you have connected Arduino to PC by using an USB port)

7. Start-up and operation

The following start-up protocol was applied to start and operate the device:

1- Open the Arduino program to control the card and configure the pin numbering, that is, identify which actuator is connected with which Arduino pin.

2- Open a Linux console and configure the USB port communication as described in Supplementary information to avoid communication issues.

3- Open Python within the console and execute the data receiving script.

4- Check that the device works as expected. The setting up will start by using regular tap water bottle instead of different bottles of chemicals (NaOH, nutrient) to perform the fluid tests.

5- Disconnect first all the pumps from the fluidic circuit. Then test electric connections of equipment by executing the electric test script. The program will first activate and deactivate electric equipment units one by one to test whether they are working properly or not. Check that every activated pin lead to the activation of the expected actuator. After that, the program will follow gathering an OD reading. Check that lecture appears in the PC screen as they are gathered. If not, first inspect and look for deficient electric connections, wiring issues and finally code bugs. Replace the actuator / component if problems still persist.

6- Once all the components are working, reconnect pumps to the fluidic circuit to check that program protocol works as expected. Run the protocol_check program, which perform an entire loop of the cycle to be repeated through the whole experiment, but using shortened times to result into a faster test. Check that liquid flows during the different stages in the correct direction, and that equipment synchronization works as expected. If liquid dynamics fails, identify in which stage the system is not working properly and try to find the bug in the program.

Once the device executes a whole iteration of the protocol without experiencing any issue, start preparing the device:

7- Autoclave H_2O (filled with distilled water) and nutrient vessel (empty), culture chamber, auxiliary vessel and humidity chamber (filled with 400 ml of distilled water). Connect all the components to the circuit.

8- Run the sterilization cycle and wait that all the system is cleaned with NaOH and washed with water.

9- Run the sample loading program and inject under sterile conditions 20 ml of culture at OD_{600} = 0.1 (previously incubated from a 3 ml overnight inoculum) within the chamber with a sterile 25 ml Luer compatible syringe. Use the injection port to inject the sample

10- Load and run the experimental protocol of interest.

11- Let the experiment running for 45 days. Take care of the stock levels. Refill them using the loading ports as described in the Supplementary information.

12- Extract 2 ml of culture through the injection port every week and prepare a glycerol stock with it to keep the population diversity. Perform contamination test of gathered samples by streaking them on LB and M9 minimal medium + 0.2 % (w/v) citrate agar plates: growing colonies in both type of plates should resemble a similar aspect.

8. Auxiliary hardware manufacturing

In this manuscript it is presented a lay-out to perform a specific protocol of interest. Most of the materials can be directly purchased directly from supplier. However, there are some other equipment that are not available to be bought, or they do not match with the chosen material. To cope up with this issue, it is proposed the use of 3D printing technology to create such components. We used a 3D printer model Ultimaker 3 with a two-nozzle head. Printing parameters used to create the designs for different used materials are provided in Supplementary Table S2.

Supplementary Table S2: Printing parameters used to manufacture the 3D printed supports and equipment

PARAMETERS	ABS	PLA	PETG	SUPPORT	unit
Layer Height	0.1	0.1	0.08	0.08	mm
Infill density	30	30	100	30	%
$T_{printing}$	265	205	245	240	ºC
T_{plate}	100	100	70	70	ºC
Print Speed	60	70	70	70	mm/s

Fan Speed	5	100	0	100	%
Support	ON	ON	ON	ON	

8.1. Auxiliary Scaffolds

It is necessary to recall that the device is a physical object that occupies space and should be properly placed in order to minimize design (wiring, tubing, etc.) and operation issues (malfunctioning, maintenance, debugging, etc.). A set of scaffolds were used to support the whole set of pumps and valves and hold the device. CAD planes are provided next in Supplementary Figures S16, S17 and S18. CAD planes are also provided with supplementary information to allow the reader to customize the designs according to their needs. The manufacturing of all supports was performed using PLA or ABS, using as main printing parameters the ones reported in Supplementary Table S2.

8.2. OD_{600} chamber

The assembly of a cyclic log-phase batch fermentor required the implementation of a sensor to monitor the optical density of the culture. In the present case study, we proposed the manufacturing of a 3D printed OD_{600} chamber. The chamber consisted on three parts: the wet chamber where the reading takes place, a scaffold to support it and the electronic hardware.

8.2.1. Wet chamber

A wet rectangular measurement chamber with a 5 mm optical step was manufactured using PETG for the structure and SUPPORT material for the cavities in the design. Blueprint design is provided in Supplementary Figure S19.

After manufacturing, support material was removed from the printed piece by dissolving it using limonene. The chamber was further treated with a dichloromethane vapor bath (using a glass beaker and heating it at 50ºC) for 3 minutes into a laminar flow cabin (Tsuda, et al., 2015). Finally, the chamber sides were sealed with 0.5 mm transparent PETG membranes by using silicon glue, leaving a curing time of 24 h. Before using it, perform a leaking test by introducing tap water inside the chamber, close the sides and leave it at rest. Wait at least 12 h and look for any possible water spill during that period.

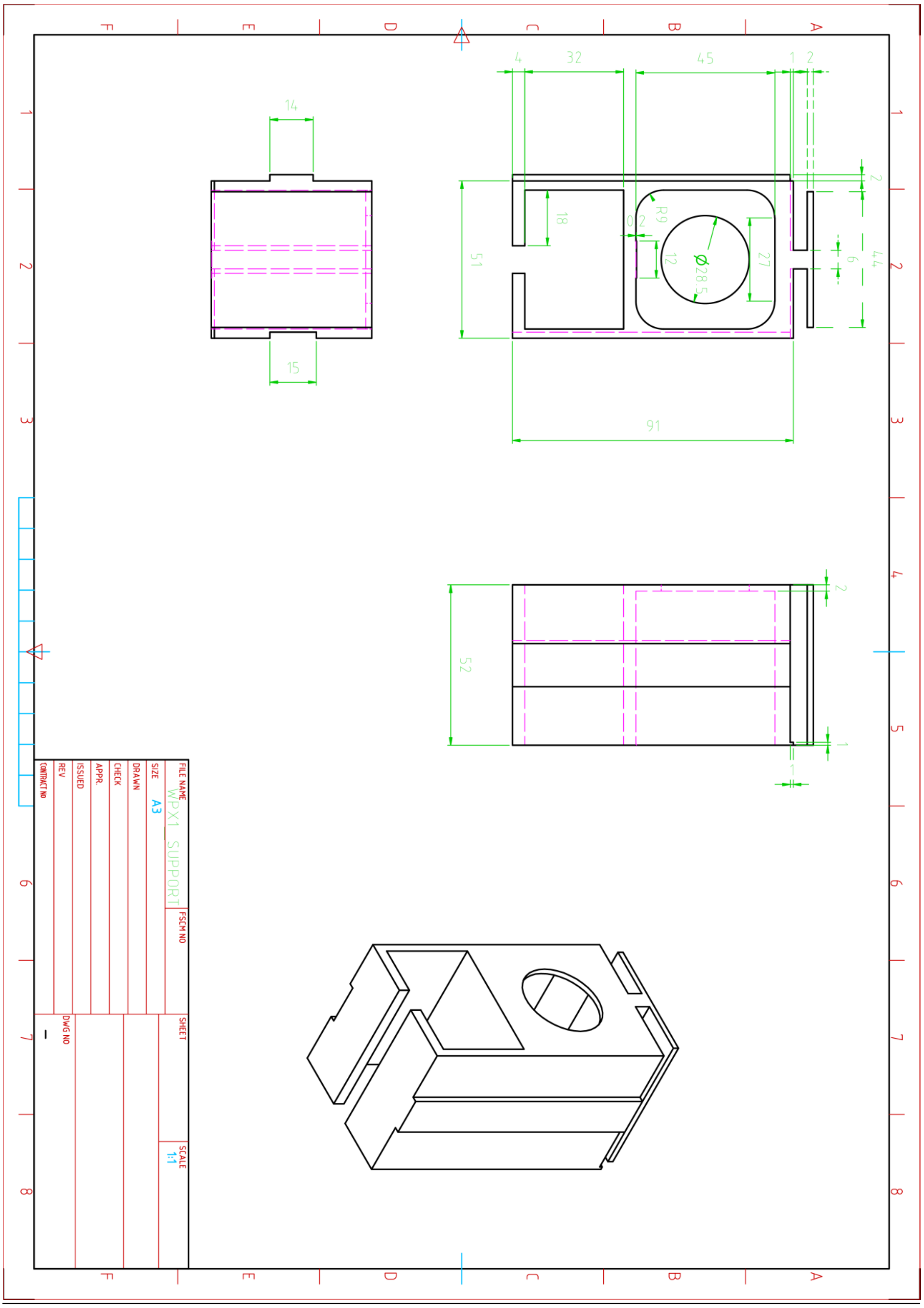

Supplementary Figure S16: Drawing showing for WPX1 pump support design

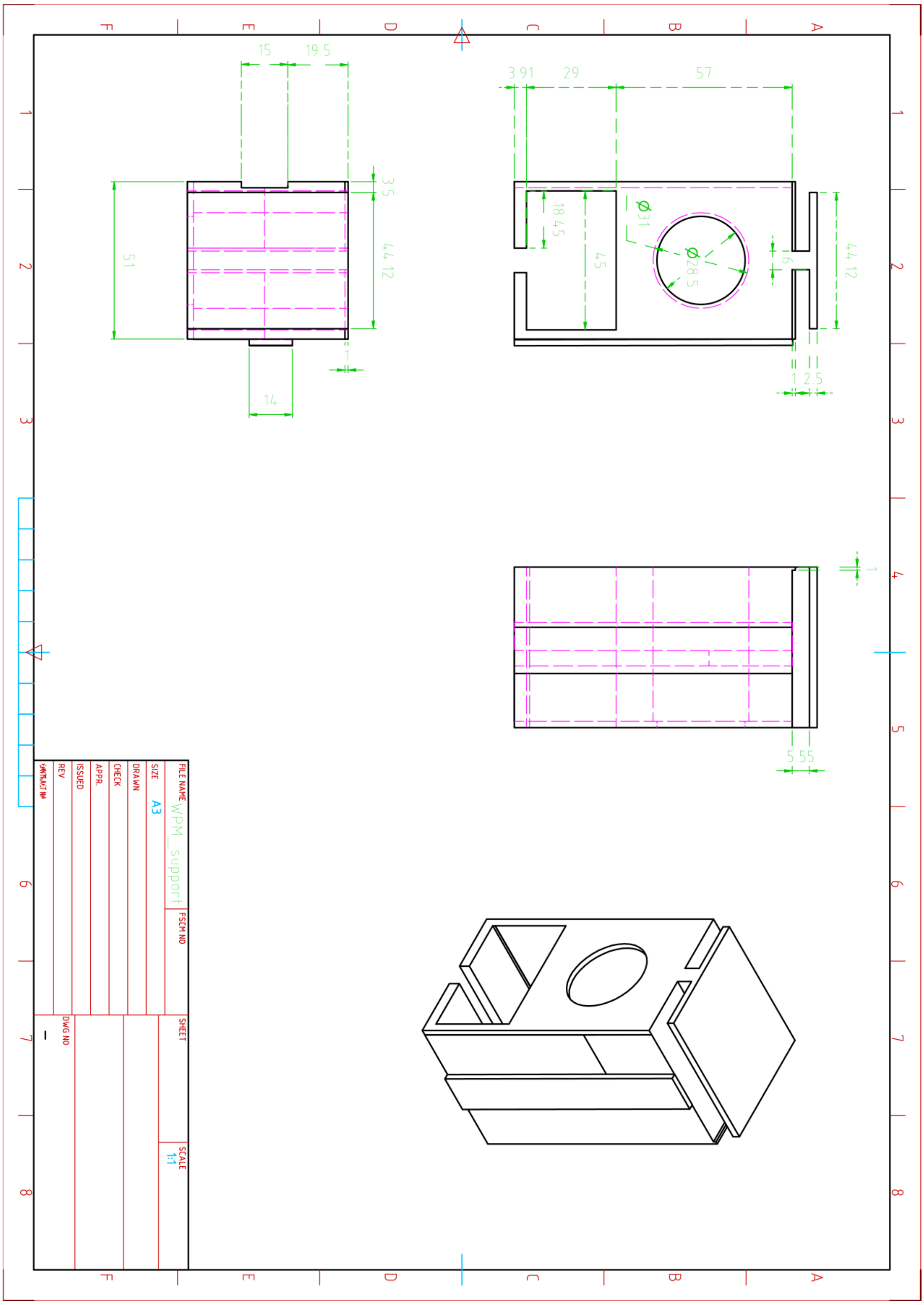

Supplementary Figure S17: Drawing showing WPM pump support design

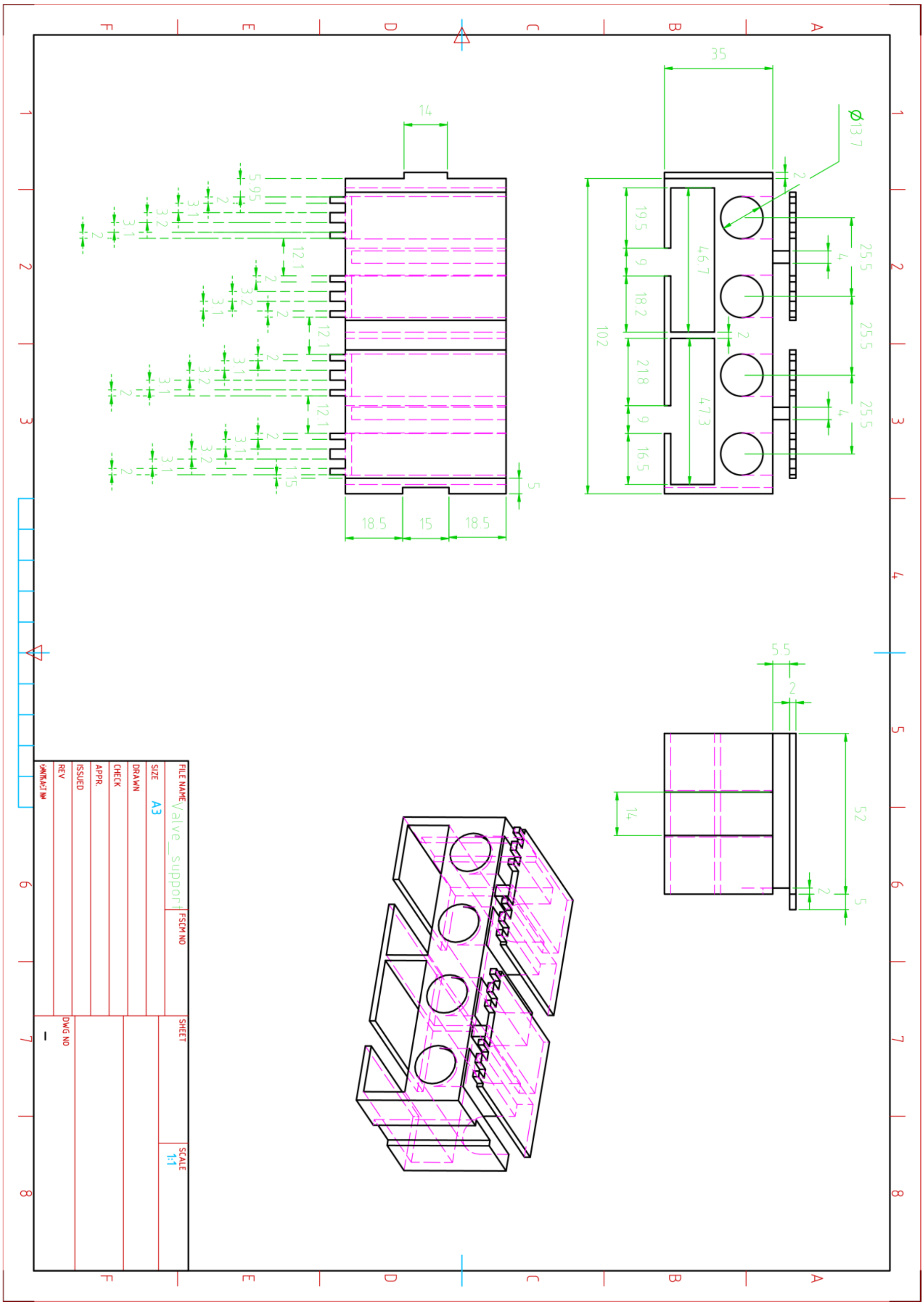

Supplementary Figure S18: Drawing showing valve scaffold design

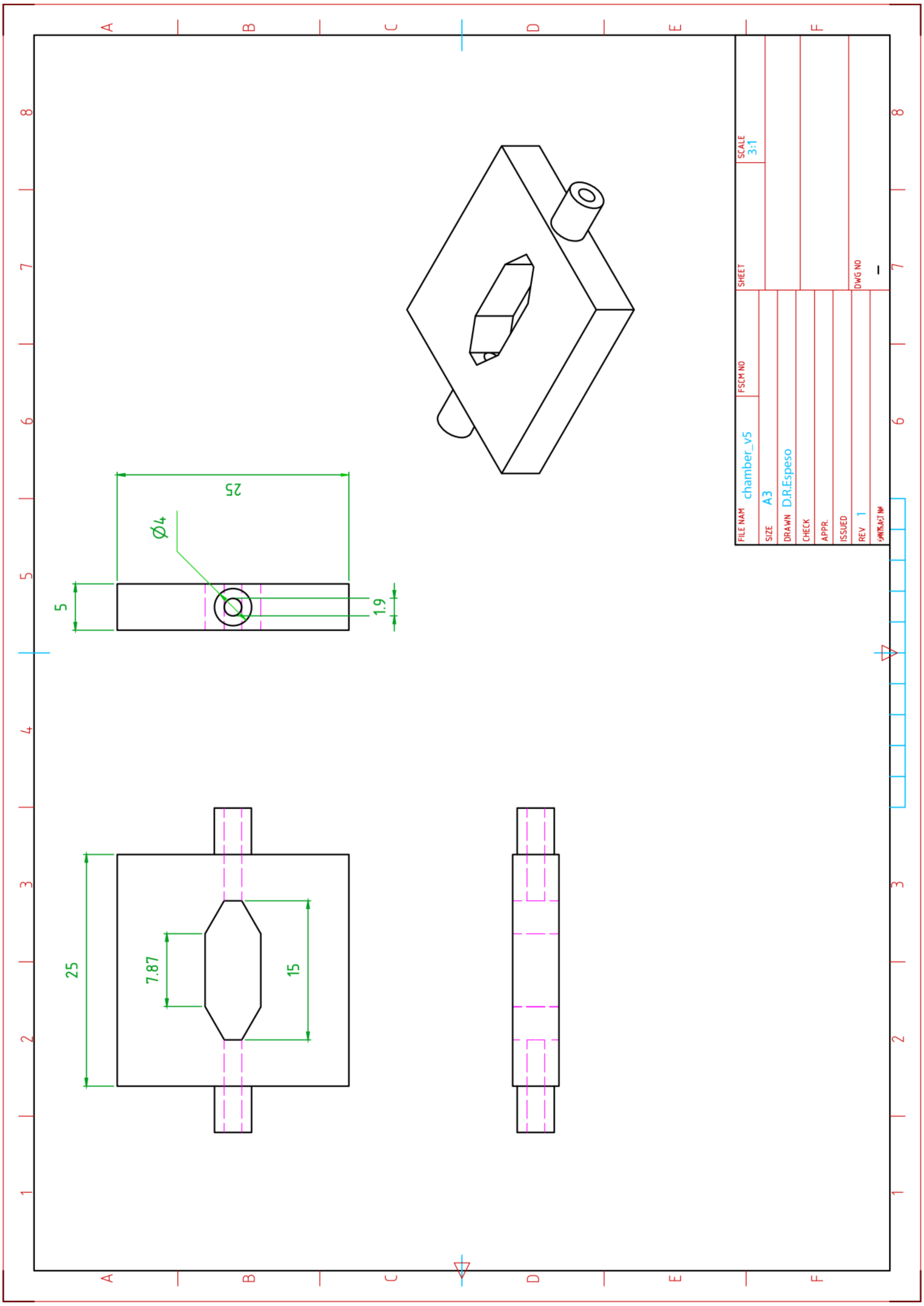

Supplementary Figure S19: Design drawing of the wet chamber

8.2.2. Chamber scaffold

The scaffold was designed to fulfill two objectives: support the electronic required to obtain the reading and cover the wet chamber to prevent noise in the light sensor readings due to environmental light. The scaffold was manufactured using black PLA. The design drawings and some images showing the assembly are included in Supplementary Figure S20.

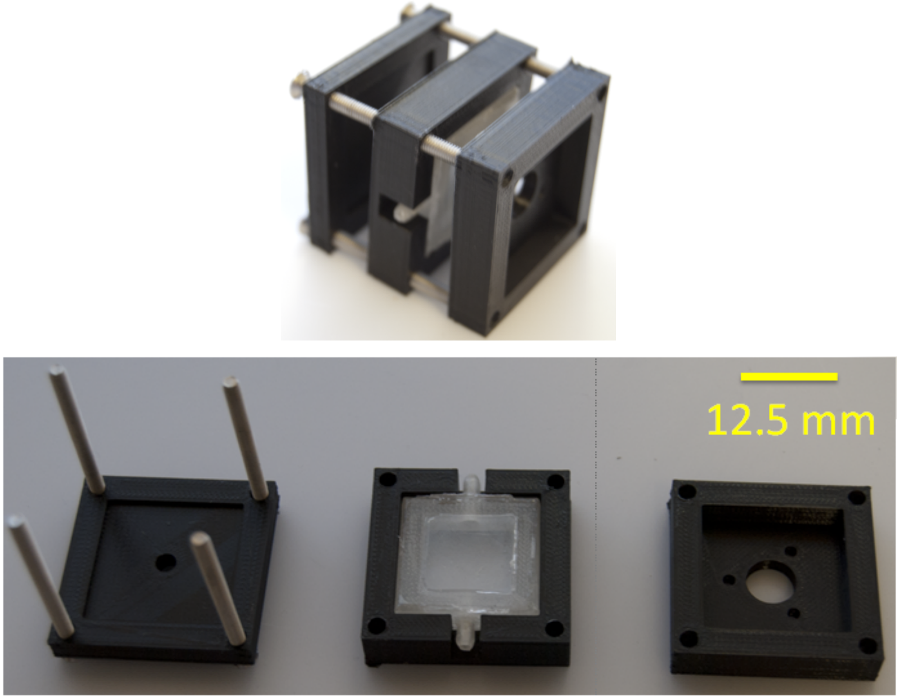

Supplementary Figure S20: Images of the optical reader set. Individual parts (down) are screwed with 4x 25 mm M3 bolts and nuts to assemble a compact closed black box with the wet chamber enclosed in it (up). Only two side holes used to emit 610 nm light and to perform the light measurements.

Blueprints of the different parts are provided next (see Supplementary Figures S21, S22 and S23)

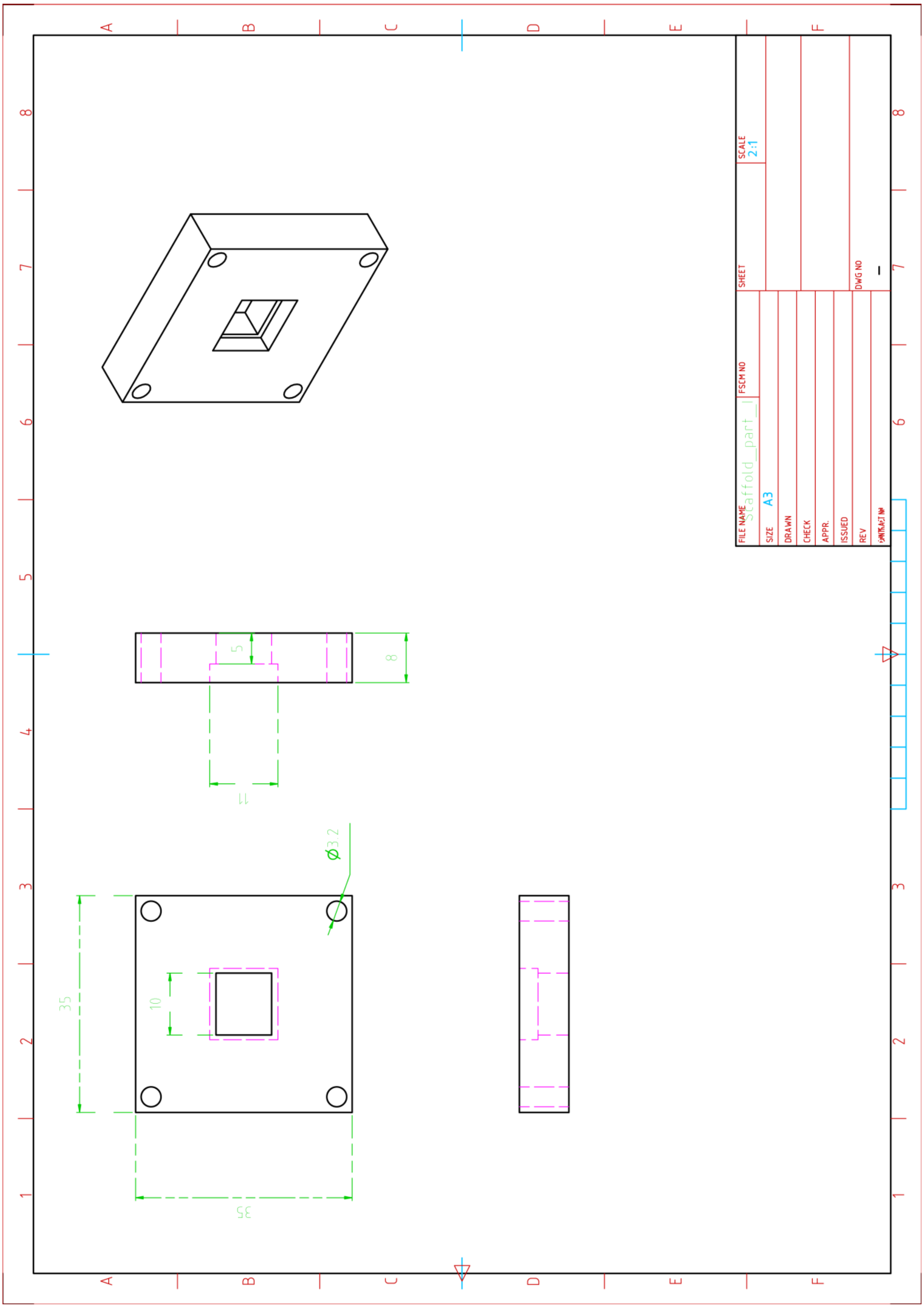

Supplementary Figure S21: Design drawing of the chamber scaffold, part I

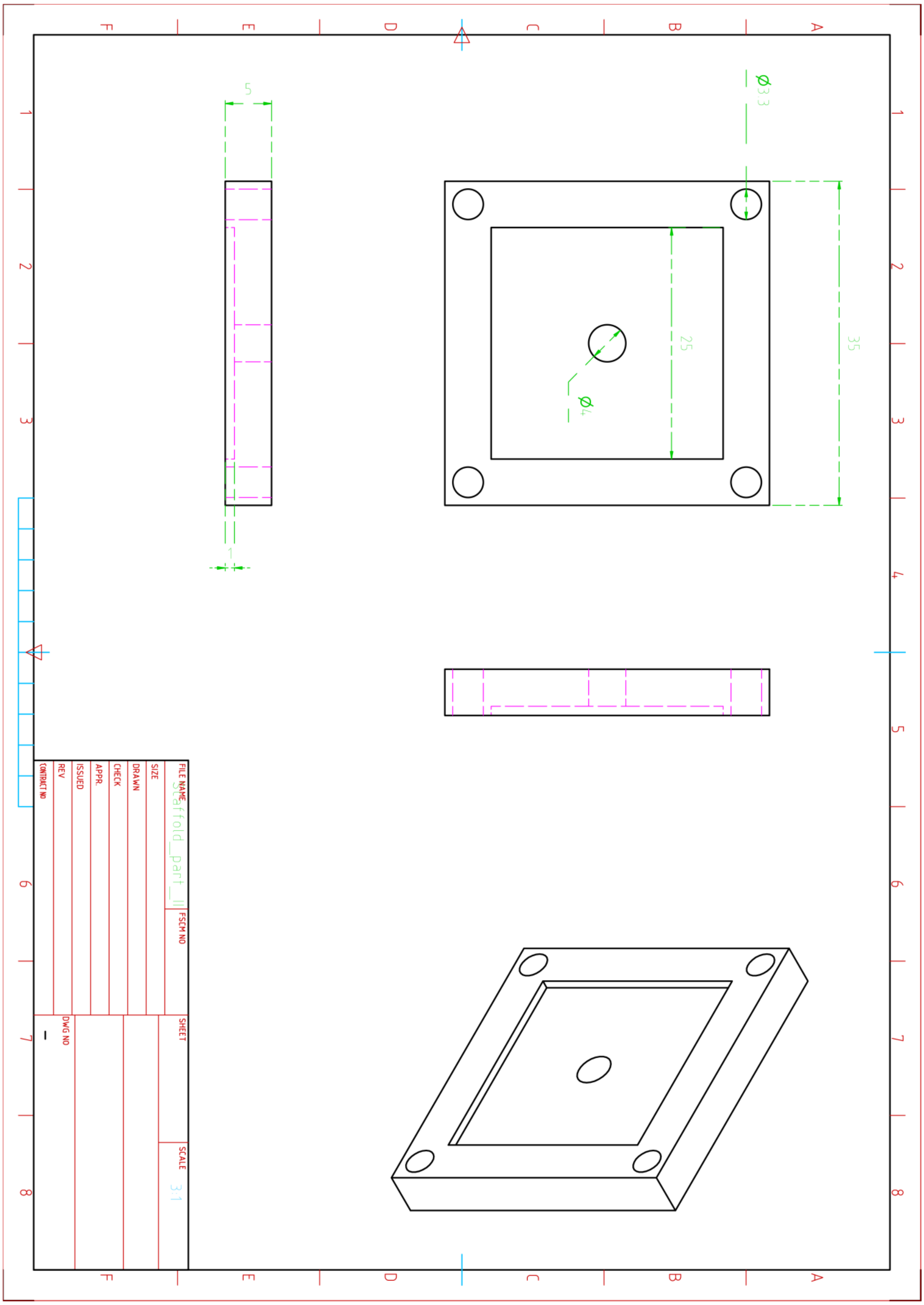

Supplementary Figure S22: Design drawing of the chamber scaffold, part II

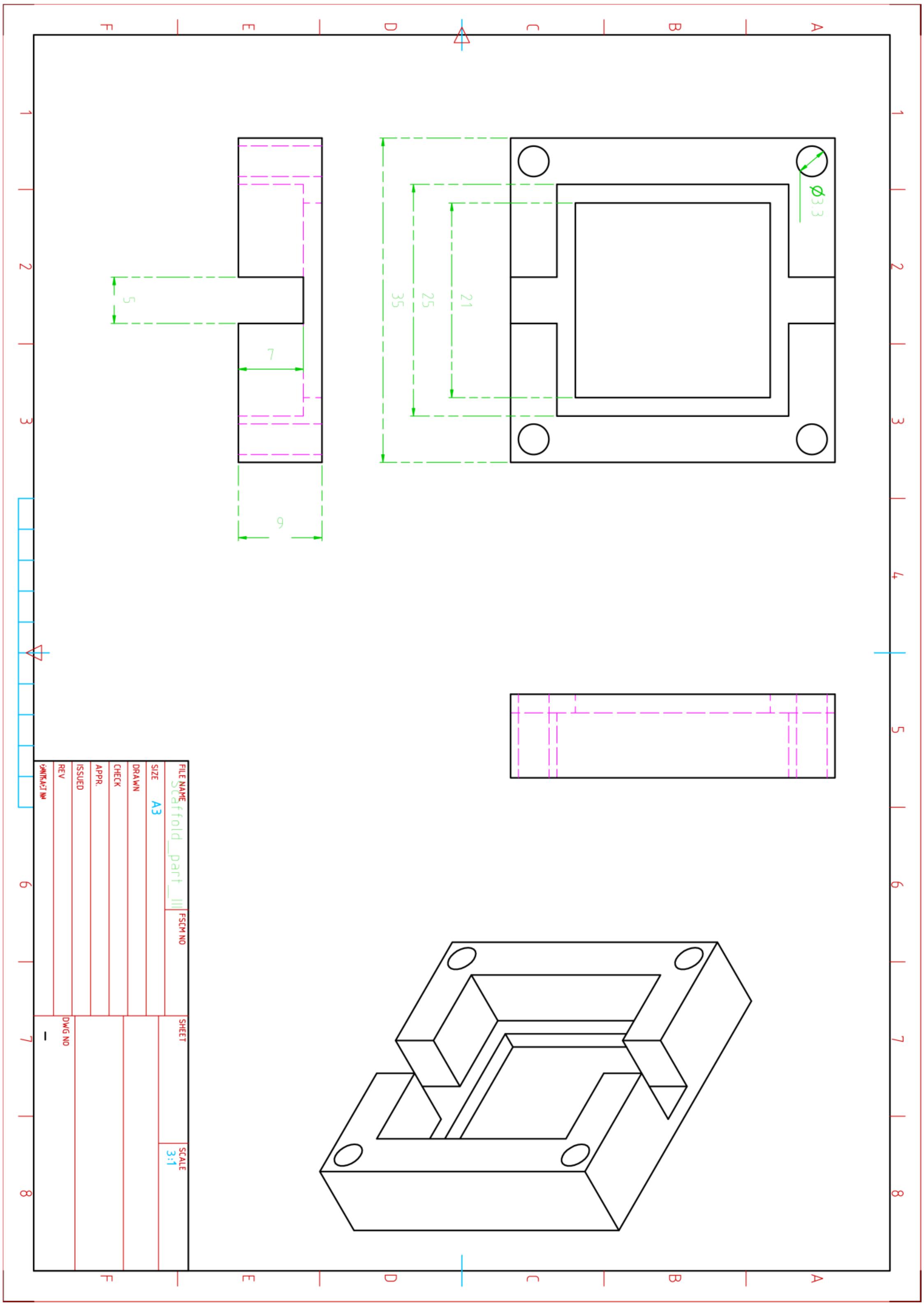

Supplementary Figure S23: Design drawing of the chamber scaffold, part III

8.2.3. Optical density reader electronic hardware

The sensor consisted on two parts: a 5 mm outer diameter LED emitting at 610 nm wavelength and a visible light sensor. The sensor and the led were aligned to allow the light crossing the chamber to measure light reduction due to bacterial presence.

Electronic wiring is straightforward: use a Fuse PCB (or a similar model) and solder in one side a KK254 switch, and in the other solder the LED (bewaring the polarity of the LED). Solder in the center of the PCB a 330 Ω. Finally connect the light sensor with a wire headed to a three way female KK254 connector.

8.2.4. Calibration and testing

Prior to use, an optical density vs detected light signal calibration slope in the range OD_{600} = [0,1] was performed to correlate both variables. The optical density sensor was also tested with an overnight growth test.

Briefly, an overnight *P. putida* KT2440 preinoculum prepared from a frozen stock in a 20 ml volume (in a 100 ml Erlenmeyer) of M9 minimal medium supplemented with 0.2% (w/v) glucose. A set of culture samples (5 ml in test tubes) with OD_{600} values ranging from 0 to 1 were prepared and the respective light measurements were gathered. A calibration slope was created to fit light readings with optical values of the samples (see Supplementary Figure S24).

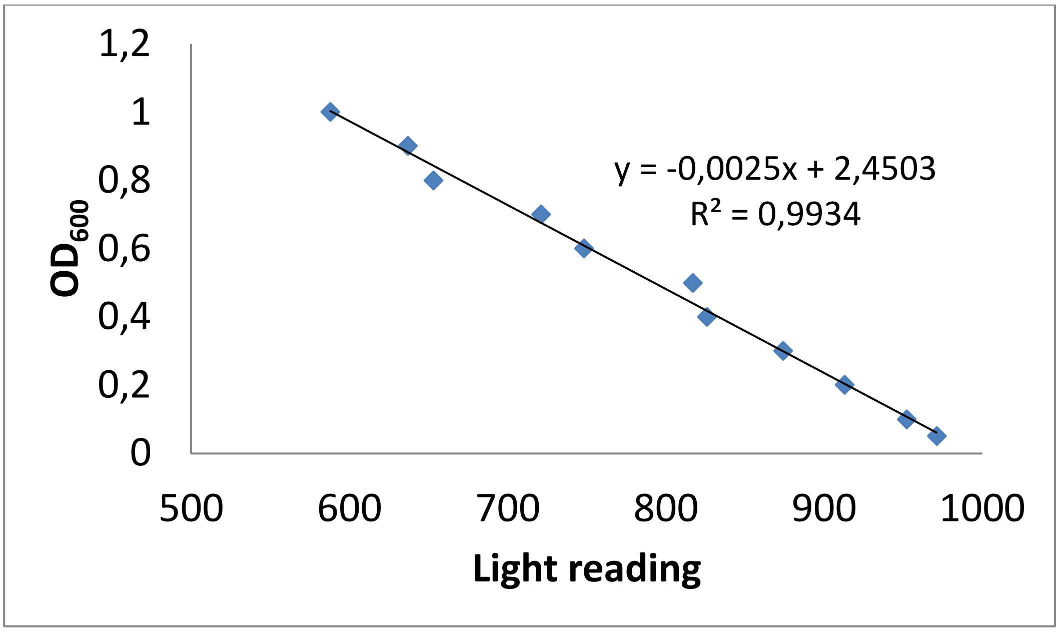

Supplementary Figure S24: Calibration slope relating light reading vs optical density in a *P. putida* KT2440 culture grown in M9+0.2% (w/v) glucose using the propose custom-made optical reader.

Once the calibration slope was ready, a second sample of culture was diluted to OD_{600}= 0.05 in 20 ml (into a 100 ml flask) of the same nutrient medium and incubated overnight. The cultured was aerated and shaken by bubbling using an air compressor pump. During every reading, a small liquid volume was transported into the wet chamber using a peristaltic pump, and returned to the culture flask after the reading was gathered. Light lectures were gathered every 5 minutes. Registered OD_{600} lectures are depicted in Supplementary Figure S25.

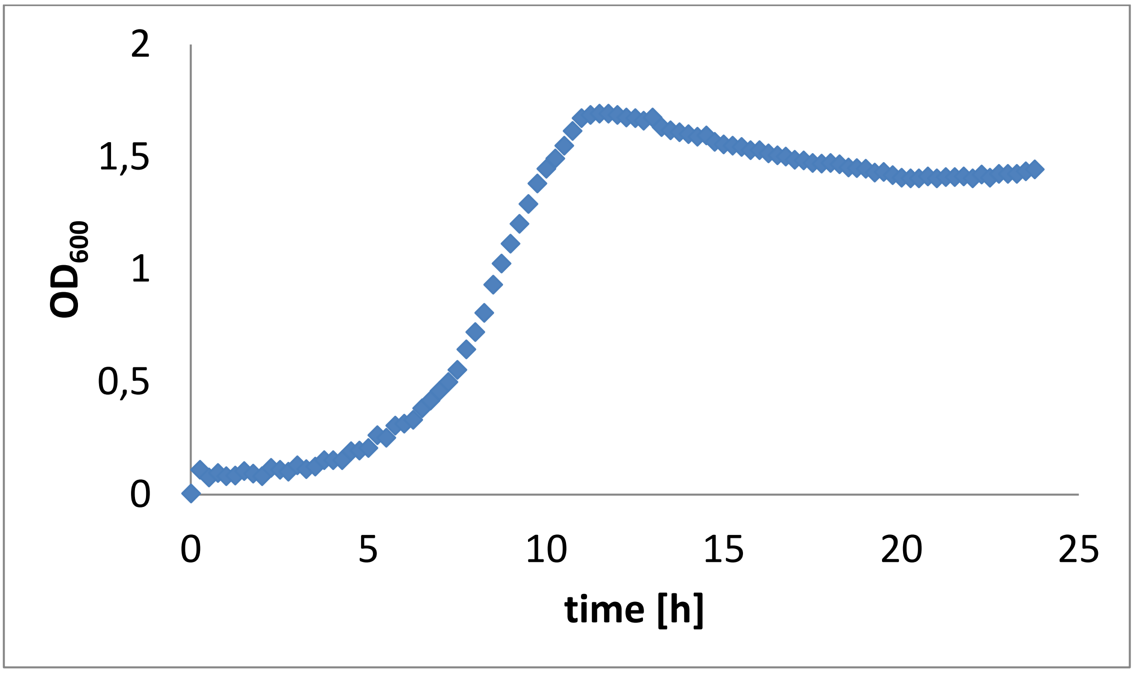

Supplementary Figure S25: OD_{600} readings obtained during the overnight incubation of *P. putida* KT2440 culture grown in M9+0.2% (w/v) glucose using the device.

As it can be observed, the curve follows a typical exponential and saturation growth behavior typically observed in this kind of experiment. The observation of the exponential part of the curve shows a maximum growth rate around 0.376 h^{-1}, which is close to the reported value for this strain (Nikel, et al., 2015). The difference of values may be caused because of the different shaking mechanism used to incubate the culture (bubbling vs orbital shaking).

8.2.5. Supervision and issues

Optical density sensor is quite sensible to small variations in light exposition when operating. If the assembly of the emitting LED or light sensor is not robust enough, it might lead to numerical drift in obtained lectures. Supplementary Figure S26 shows the growth rate evolution estimated from optical density readings during the performed experiment. At day 30 an unexpectedly misalignment caused by an improperly assembling process led to the detection of overestimated lectures, which boosted the values of detected growth rate. As a consequence, the culture was repeatedly diluted till OD_{600} values below 0.1 (as seen in Supplementary Figure 2B in the main manuscript). Nevertheless, when the sensor was properly aligned, the culture start increasing the correct operation densities and growth rate values were reestablished to previously detected values. This kind of events cannot be predicted and might ruin an experiment; thus, researchers are warned about checking periodically that optical density lectures displayed on the screen are within the expected values. On the other side the appearance of beneficial mutation events, although hardly expected, are still possible during an experiment. These changes might cause extraordinary increases in growth rate, being possible to generate a very similar effect in the optical density readings. The researcher should always double check that such abrupt changes are not caused by a hardware / software issues but is a result of the evolution process.

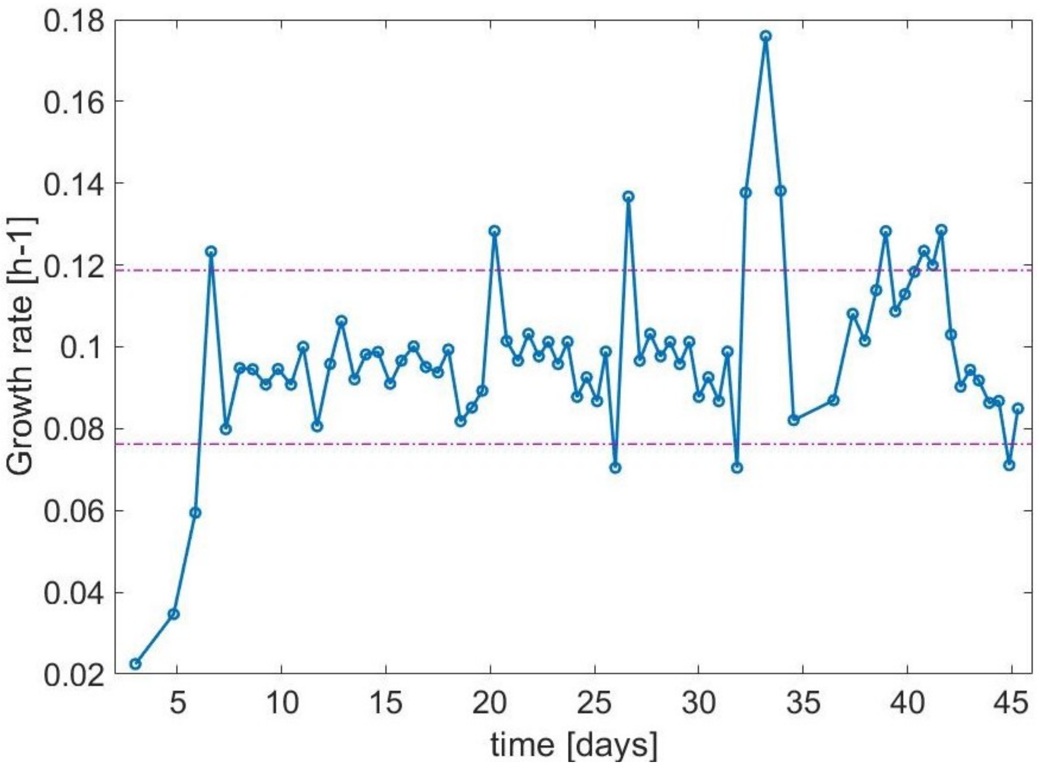

Supplementary Figure S26: Growth rate evolution during the experiment development. The sharp peak located at day 30 was a result of a numerical drift caused by the detection of arbitrarily larger values of optical density lectures due to a hardware failure. Once realigned, the optical sensor started working properly. Dashed lines mark the average value ± standard deviation.

Another issue to take into consideration when designing the automated operation protocol of the device is the accumulation of small drifts in relevant measurements. Here, we spot a source of error detected when designing the automated protocol, which affected the OD_{600} data. As can be seen in Fig 2, the initial OD_{600} after every dilution step did not show a constant value during the experiment (as it was expected to happen) but tended to slightly increase over time. The reason for such behavior was the presence of an operational time lag between the OD600 reading and the effective dilution of the culture (which was around 30 minutes, the duration of the main chamber washing time). When designing the protocol, this period of time was not considered as effective incubation time. This fact led to unexpected increments in the actual optical density values when dilution was about to happen every iteration. As a consequence, all dilution steps were performed in base of underestimated OD_{600} lectures. Although these mistakes were very small when considering in a single step (at the beginning of the experiment, the increase of OD_{600} could not be detected by the sensor due to the very low growth rate of the template strain), the large number of dilutions performed during the whole experiment permitted an accumulation and amplification of the error, generating the observable drift in the optical density curve.

8.3.

9. Template strain preparation

Supplementary Table S3: Strains and plasmids used in this study

Strain or plasmid	Characteristics	Source or reference
Escherichia coli		
CC118λ*pir*	Cloning host: *araD139* Δ*(ara-leu)7697* Δ*lacX74 galE galK phoA20 thi-1 rpsE rpoB*(Rif^R) *argE*(Am) *recA1*,λpir lysogen	(Herrero, et al., 1990)
Pseudomonas putida		
KT2440	Wild-type strain, spontaneous restriction-deficient derivative of strain mt-2 cured of the TOL plasmid pWW0	(Bagdasarian, et al., 1981)
EM42 Δ*gcd*	Derivative of *P. putida* KT2440: Δprophages1,2,3,4 ΔTn7 Δ*endA1* Δ*endA2* Δ*hsdRMS* Δflagellum ΔTn*4652* with scarless deletion of *gcd* gene (PP_1444) encoding periplasmic glucose dehydrogenase	(Dvořák and de Lorenzo, 2018)
mk-1 (EM42 Δ*gcd::Pem7-xylABE-aadA*)	EM42 Δ*gcd* with insertion of synthetic expression cassette containing constitutive EM7 promoter, synthetic *xylABE* operon, T500 terminator and *aadA* with own regulatory sequences.	This study
**Plasmids**		
pBAMD1-4_P_{EM7}	Derivative of original pBAMD1-4 mini-Tn5 delivery plasmid: *ori*(R6K) *bla aadA*, Amp^R Sm^R/Sp^R with constitutive EM7 promoter (*Avr*II/*Eco*RI).	Gift from Dr. Alberto Sánchez-Pascuala
pSEVA2213_*xylABE*	Low copy expression vector: *oriV*(RK2) P_{EM7} *neo*, Km^R with *xylAB* part of *xyl* operon from *E. coli* encoding XylA xylose isomerase and XylB xylulokinase (*Eco*RI/*Bam*HI) and *xylE* gene from *E. coli* encoding XylE xylose-proton symporter (*Bam*HI/*Hind*III).	(Dvořák and de Lorenzo, 2018)
pBAMD1-4_P_{EM7}-*xylABE*	Derivative of pBAMD1-4_P_{EM7} with synthetic xylABE operon (*Eco*RI/*Hind*III).	This study.

Abbreviations: Amp, ampicillin; Km, kanamycin; Sm, streptomycin; Sp, spektinomycin.

9.1. Preparation of *P. putida* strain with chromosomal insertion of *xylABE* operon

The synthetic *xylABE* operon (Dvořák and de Lorenzo, 2018) encoding XylA xylose isomerase, XylB xylulokinase, and XylE xylose-proton symporter from *Escherichia coli* BL21(DE3) was subcloned from pSEVA2213_*xylABE* construct into *Eco*RI and *Hind*III restrion sites of Mini-Tn5 delivery plasmid pBAMD1-4 (Martínez-García, et al., 2014) with constitutive EM7 promoter cloned between *Avr*II and *Eco*RI sites of standard SEVA polylinker. The

pBAMD1-4_P_{EM7}-*xylABE* construct was propagated in *E. coli* CC118 *λpir* cells and 200 ng of purified plasmid were used for transformation of *Pseudomonas putida* EM42 Δ*gcd* strain (Dvořák and de Lorenzo, 2018) using electroporation protocol described previously (Martínez-García and de Lorenzo, 2012). Cells were recovered for 7 h in Terrific Broth without glycerol (per 1 l: 12 g tryptone, 24 g yeast extract, 100 ml of 0.17 M KH_2PO_4 and 0.72 M K_2HPO_4 solution) at 30°C. Next, cells were spinned at 4000 rpm for 10 min, washed once with M9 minimal medium (per 1 l: 8.5 g Na_2HPO_4 $2H_2O$, 3.0 g KH_2PO_4, 0.5 g NaCl, 1.0 g NH_4Cl, added with $MgSO_4$ to the final concentration of 2 mM, and with 2.5 ml/l trace element solution prepared based on (Abril, et al., 1989)) and resuspended in 100 ml of the same medium with 10 g/l D-xylose and streptomycin (50 µg/ml) to the starting OD_{600} of 0.25. Cells were incubated in the selection medium at 170 rpm (30 °C) for four days. Cells were then centrifuged at 4000 rpm for 10 min and plated at appropriate dilutions on M9 agar plates with 5 g/l D-xylose and streptomycin (60 µg/ml). After 4 days of incubation at 30 °C, hundreds of faster growing colonies of approximately same size were observed on each plate on background of thousands of tiny slowly-growing colonies. Three faster growing colonies were re-streaked on fresh M9 agar plate with xylose and glycerol stocks in 1 ml of LB medium with 20 % glycerol were prepared and stored at – 80 °C for further use.

Position of P_{EM7}-*xylABE-aadA* cassette (*aadA* encodes aminoglycoside resistance protein) in chromosome of each of the three clones was determined by arbitrary PCR using the GoTaq Green Master Mix (Promega) and protocol described by (Martínez-García, et al., 2014). Primers Arb6 and ME-O-Sm-Ext (all primer sequences for arbitrary PCR can be found in (Martínez-García, et al., 2014)) were used in the first reaction of total volume of 25 µl. Small portion (1 µl) of the first PCR mix was transferred in the second reaction of 50 µl volume with primers Arb2 and ME-O-Sm-Int-F. PCR products were purified using NucleoSpin Gel and PCR clean-up kit (Macherey-Nagel) and sent for sequencing with ME-O-Sm-Int-F primer (Macrogen). Obtained sequences were used as queries for BLASTN search in *P. putida* KT2440 chromosomal sequence on Pseudomonas.com server. Identified position of insertion was further verified in each of the three candidates by colony PCR with GoTaq Green Master Mix (Promega) and primer pair ME-O-Sm-Ext and PP2260 check rv (GCTGTCGAAAACGAACAGCTTGTGCG) giving rise to the PCR product of about 700 bp.

9.2. Viability of the recombinant strain

It has been shown before that expression of the three genes *xylABE* in the synthetic operon allows growth of the host bacterium in minimal medium with D-xylose as a sole carbon and energy source (Dvořák and de Lorenzo, 2018) . The fastest growing exconjugant clones of *P. putida* EM42 Δ*gcd* with the *xylABE* insert happened all to have the synthetic catabolic cassette inserted in the same chromosomal location position. This was the gene encoding a putative cytoplasmic-membrane associated glycerol phosphate ABC transporter ATP-binding protein (PP_2260, coordinates 2578591-2579685). In view of this, only one clone was selected for bioreactor experiment (named in the manuscript as *mk-1*). To ensure that the insertion was nor detrimental for or general performance of the thereby modified strain recombinant strain, it was compared to the precursor strain EM42 Δgcd in either rich LB medium or M9 minimal medium with glucose or citrate. Strains mk-1 and EM42 Δ*gcd* (control) were inoculated from glycerol stocks in 2.5 ml of LB medium (in case of the former strain with streptomycin 60 µg/ml) and grown overnight at 30°C with shaking (170 rpm). Cells were briefly spinned at 4000 rpm for 5 min, washed with fresh LB medium (per 1 l: 10 g tryptone, 5 g yeast extract, 10 g NaCl) or with M9 minimal medium and re-suspended in 150 µl of LB or M9 minimal medium with 5 g/l citrate or 5 g/l glucose in the wells of transparent 96-well microtiter plate with flat bottom to the starting OD_{600} of 0.1. Cells were grown in Victor[2] 1420 Multilabel Counter (Perkin Elmer) at 30°C and optical density in the individual wells was measured at 30 min intervals.

The results (Supplementary Figure S27) suggested that both strains were virtually indistinguishable: No effect of chromosomal insertion on viability in neither of the media was observed.

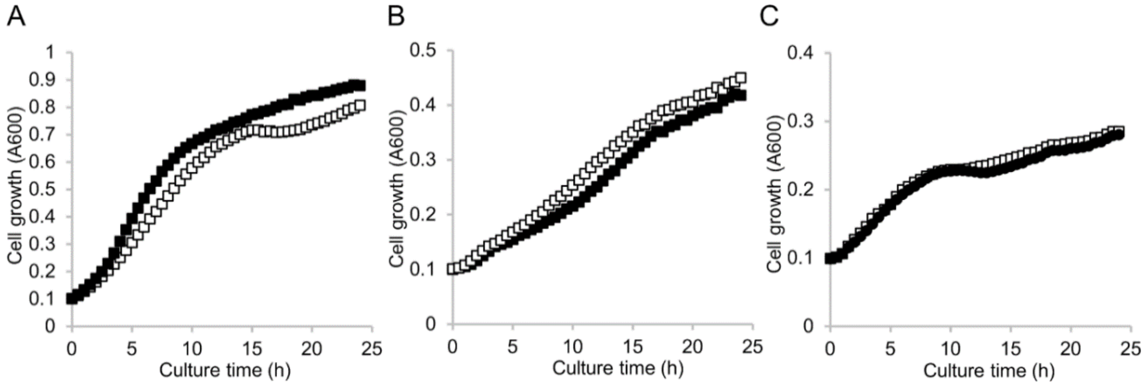

Supplementary Figure S27. Comparison of growth of *P. putida* EM42 Δgcd (control) and mk-1 strain in rich growth medium and in minimal medium with carbon source. A) Rich lysogeny broth medium; B) M9 minimal medium with 5 g/l glucose. C) M9 minimal medium with 5 g/l citrate. Experiment was carried out in microtiter plate (150 µL of medium per well) at 30°C. *P. putida* EM42 Δgcd, filled squares; mk-1, open squares. Data points shown as means of absorbance A_{600} of four biological replicates. Standard deviations were within 10 % of the mean values.

10. ALE design constrains

As reported in the main manuscript, working with *P.putida* KT2440 or its derivative strain EM42 demanded modifying the experimental setup to satisfy certain constrains. They are listed below:

- *P. putida* KT2440 strain naturally generates considerable amount of biofilm onto surfaces. Biofilm formation is undesirable not only from an operative point of view (it generates clogging issues and reduces the efficiency of all fluidic actuators and sensors) but also for the final objective of obtaining an improved phenotype: bacteria under biofilm shape are less exposed to the pressure selection (liquid dilution), delaying or even halting the appearance of improved phenotypes. Avoid biofilm formation was considered a priority.
- *P. putida* KT2440 is strictly aerobic. It was necessary to ensure a proper aeration during the whole experiment.
- *P, putida* mk-1 was poorly fitted to growth using D-xylose as carbon source. Other environmental microorganism could contaminate the experiment if hardware was not properly designed. Device design must ensure isolation of the manipulated culture by design.
- The suboptimal growth of the template strain, which was approximately three-times slower than the growth reported for *P. putida* EM42 ∆gcd with xylABE operon on pSEVA2213 plasmid (Dvořák and de Lorenzo, 2018), suggested metabolic issues related to low expression of pathway genes from single copy in chromosome, or compromised cell viability caused by chromosomal insertion. Since growth experiments performed with mk-1 strain and *P. putida* EM42 ∆gcd control proved minimal or no effect of insertion on cell viability, suboptimal pathway expression was considered to be the major cause of the slow growth. It was a priority to maximize cell division by ensuring growth media quality (i.e. pH stability, absence of debris or metabolic by-products, etc.) to boost the generation of phenotypic diversity in the culture.

Taking into consideration all these constrains, several design features were included in the device design:

- The system required the implementation of a periodic washing protocol to get rid of biofilm formed on the chambers and the whole fluidic device. This was achieved by introducing an auxiliary chamber to contain the culture between cleaning programs, including stocks of cleaning agent (sodium hydroxide, 2M) and

sterile water. Tubing exposure to bacterial culture or chemicals was minimized by pumping filtered air after every liquid handling operation.

- Culture was constantly aerated by bubbling filtered humid air into the culture. This ensured the presence of oxygen in the liquid, its mixing and an additional barrier to outer contamination due to positive pressure inside the culture chamber. To check whether this method of aeration would be enough to allow the culture growing under aerobic conditions, an approximate estimation of the required rate of oxygen required to operate within this regime was calculated. Concretely we characterize the typical oxygen mass transfer parameter product (k_La), and then the oxygen transfer rate (OTR) under the chosen experimental conditions with the correlations found in (Büschs et al., 2000; Humbird et al., 2017). Considering only a reduced amount of the maximum flow rate (to avoid issues such as stressing cells due to excessive mechanical stirring or to avoid the formation of foam), we obtained an OTR value of 2.41E-3 mmol/L·s. When this value was compared with the estimated specific consumption rate of D-xylose of *P. putida* EM42 see (Dvorak and de Lorenzo, 2018) (which was around 3.7 mmol/L·s for an OD_{600} average of 0.4), we observed a 65-fold change. Having into account that biomass production – oxygen consumption stoichiometry would be quite far from this value, it meant that oxygen would be in excess within the culture at any time of every growth cycle.

- The nutrient solution used to incubate cells was supplemented with antibiotic (60 µg/ml streptomycin) to minimize risk of contamination with other bacteria.

11. Other procedures

11.1. Stock refilling

Stock refilling is an operation that involves replenishment of chemicals used by the fluidic layer to operate. In the presented case study, three chemicals are used: NaOH 2M, H_2O and nutrient source (M9+0.2 % D-xylose). Refilling sodium hydroxide can be performed in a straightforward fashion because no biological agents can grow in it. However, distilled water and nutrient are susceptible contamination. In this framework, we propose a refilling system without replacing the connected flask to the circuit, minimizing the risk of contamination when manipulating (see Supplementary Figure S28). It consists of using a loading port that transfers the liquid from an auxiliary stock bottle into the connected bottle using a sterile syringe (Supplementary Figure S28-1 and S28-2).

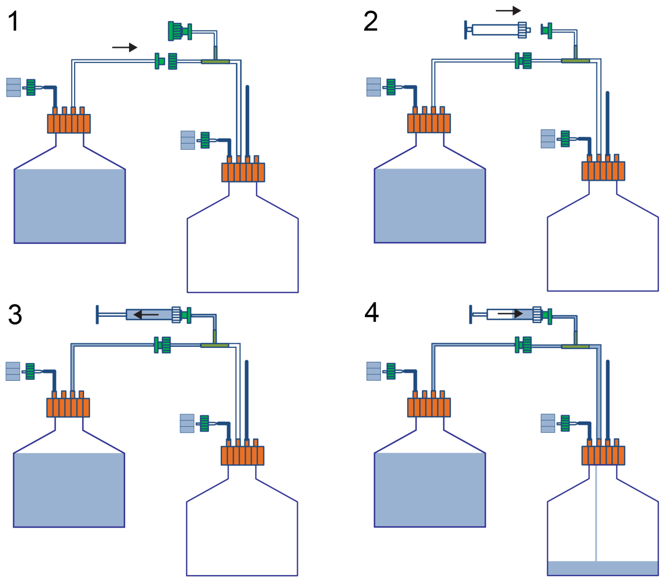

Supplementary Figure S28: Stock loading system. By using a loading port (1) and coupling a sterile empty syringe (2), the tubes connecting both bottles are filled with liquid (3), which induce a liquid transfer from the new stock bottle (left) to the empty reactor container bottle (right) by height differences (4) with minimum risk of contamination.

The syringe generates local vacuum in the loading port tubing when sucking, forcing the liquid to fill the tubing (Supplementary Figure S28-3). Once the syringe is emptied, the liquid is transferred from one bottle to the other by column height difference (Supplementary Figure S28-4). Threading and unthreading of Luer ports must be performed carefully, using ethanol to cover the connectors and the caps

before and after every loading operation. Always use aluminum foil and Luer caps to cover connector threads, thus minimizing exposition to external contamination.

11.2. Culture sampling

Loading port IP-0 can be used to extract samples from the reactor using a sterile syringe. Just wait until the reactor is in incubation stage. Then sterilize the port using ethanol before manipulation, and connect the syringe. Take the desired sample and put the cap again on the port.

12. Methods

12.1. Growth rate measurements

The evaluation of the growth rate was performed by plotting the growth curves for every strain (the evolved strain and template strain) and comparing the shape of both plots. Briefly, 100 ml Erlenmeyer flasks containing 20 ml of M9 minimal medium supplemented with 0.2% (w/v) D-xylose and antibiotics as required (60 µg/ml streptomycin) were inoculated from -80ºC frozen stocks and cultured overnight to allow cultures reaching an OD_{600} not larger than 0.5 (corresponding typically to an exponential growth state). Cultures were diluted to an initial OD_{600} of 0.04 with the same culturing medium and left to grow during a period of 30 hours, during which optical density measurements were gathered at periodic intervals of 1 h. Three biological replicates were performed and the generated cloud point resulting of all the experiments was fitted by a regression slope to obtain the estimated growth rate. Note that as the starting preinoculum was already growing at exponential stage, it was expected that microorganisms exhibited such behavior during all the experiment. So, for a culture growing at exponential regime:

$$C = C_0 \cdot 2^{\frac{\Delta t}{t_{doubling}}} \rightarrow \log\left(\frac{C}{C_0}\right) = \log(2)\frac{\Delta t}{t_{doubling}}$$

$$\log\left(\frac{C}{C_0}\right) = k \cdot \Delta t$$

where C and C_0 are the concentration of bacteria per ml at time t and time 0, Δt is the elapsed time of the experiment, $t_{doubling}$ is the time required by the bacterial culture to double its numbers and *k* is the growth rate of the culture. Measured OD_{600} values during the experiment were used to plot the last expression (instead of real bacteria concentration for C and C_0, because they are roughly correlated by a constant factor). The growth rate of every strain was given by the slope of the plotted line.

12.2. Whole genome sequencing and data analysis

P. putida mk-1 and mk-2 strains were grown overnight in 100 ml of M9 minimal medium supplemented with 0.2% (w/v) D-xylose and 60 µg/ml streptomycin (30°C/ 170 rpm). Cells were pelleted by centrifugation (15 minutes/4000 rpm) and genomic DNA was isolated with the DNAeasy® UltraClean® Microbial Kit (Qiagen). Whole genome sequencing with Illumina platform was outsourced to MicrobesNG (Birmingham, UK). Analysis of raw data quality was done using FASTQ files with FastQC tool (https://www.bioinformatics.babraham.ac.uk/projects/fastqc/). No quality issues were detected and Illumina reads were aligned to *P. putida* KT2440 genome (NC 002947.4) with ”bwa aln” and ”bwa sampe” commands using default parameters (Li and Durbin, 2010). Alignment files in SAM

format were compressed, coordinate-sorted and indexed applying, respectively, ”samtools view -bS”, ”samtools sort” and ”samtools index” commands (Li, et al., 2009). Before coordinate-sorted step, paired reads aligning at the same genomic coordinates (considered as PCR artefacts) were eliminated with ”samtools rmdup” command. Genomic deletions present in *P. putida* EM42 ancestral strain (absent in KT2440 reference) were detected as regions with no coverage in SAM/BAM files using ”bedtools genomecov -bga” (Quinlan and Hall, 2010) and parsing the output with ”grep -w 0$”. Detection of genomic changes was carried-out using ”samtools mpileup -B” and ”bcftools call -m” (Li, 2011). Relevance of the detected polymorphisms was determined with snpEff tool by establishing 500-bp regions upstream and downstream the involved gene (- upDownStreamLen 500;(Cingolani, et al., 2012). Only variants with QUAL > 30 were considered for validation of mutations. IGV v2.8.0 software was used to visualize the detected mutations throughout the sequence alignments and estimate the number of wild-type vs. mutant reads in the mk-2 sample.

12.3. Considerations about WGS results

It is also worth to mention that not only the three mutations reported but also the wild-type sequences were detected in the WGS analysis. Different sequence reads covering the same genomic region showed either the wild-type sequence or the modification, suggesting the presence of several subpopulations in the mk-2 sample. Even when the technique does not allow a precise quantification, a proxy based on the number or reads containing the sequence variants estimates that 10-30 % cells harbor each mutation. More research is needed to establish whether the three changes co-localize in the same mutants and also the relevance of the individual mutations (or different combinations of them) in the improved phenotype reported in this work. We envisage that isolation of single mutants from mk-2 population, together with a thorough characterization of the detected changes, would shed light on adaptations imposed by the ALE procedure.

13. References

Abril, M.A., Michan, C., Timmis, K.N., and Ramos, J.L. (1989) Regulator and enzyme specificities of the TOL plasmid-encoded upper pathway for degradation of aromatic hydrocarbons and expansion of the substrate range of the pathway, *J. Bacteriol.* **171**: 6782-6790.

ASTM (2015) Standard Specification for Plastic Insert Fittings for Polyethylene (PE) Plastic Pipe, *ASTM International* **D2609-15**.

Bagdasarian, M., Lurz, R., Rückert, B., Franklin, F.C., Bagdasarian, M.M., Frey, J., and Timmis, K.N. (1981) Specific-purpose plasmid cloning vectors. II. Broad host range, high copy number, RSF1010-derived vectors, and a host-vector system for gene cloning in Pseudomonas, *Gene* **16**: 237-247.

Büschs, J., Maier, U., Milbradt, C. and Zoels, B. (2000) Power Consumption in Shaking Flasks on Rotary Shaking Machines: I. Power Consumption Measurement in Unbaffled Flasks at Low Liquid Viscosity. *Biotechol. Bioeng*. **68**: 589-593

Cingolani, P., Platts, A., Wang le, L., Coon, M., Nguyen, T., Wang, L., et al. (2012) A program for annotating and predicting the effects of single nucleotide polymorphisms, SnpEff: SNPs in the genome of *Drosophila melanogaster* strain w1118; iso-2; iso-3, *Fly (Austin)* **6**: 80-92.

Dvořák, P., and de Lorenzo, V. (2018) Refactoring the upper sugar metabolism of *Pseudomonas putida* for co-utilization of cellobiose, xylose, and glucose, *Metab. Eng.* **48**: 94-108.

Herrero, M., de Lorenzo, V., and Timmis, K.N. (1990) Transposon vectors containing non-antibiotic resistance selection markers for cloning and stable chromosomal insertion of foreign genes in gram-negative bacteria, *J. Bacteriol.* **172**: 6557-6567.

Humbird, D., Davis, R. and McMillam J.D. (2017). Aeration costs in stirred-tank and bubble column bioreactors. *Biochem. Eng. J.* **127**: 161-188

ISO (2016) Small-bore connectors for liquids and gases in healthcare applications – Part 7: Connectors for intravascular or hypodermic applications, *ISO standards* **80369-7:2016**.

Li, H. (2011) A statistical framework for SNP calling, mutation discovery, association mapping and population genetical parameter estimation from sequencing data, *Bioinformatics* **27**: 2987-2993.

Li, H., and Durbin, R. (2010) Fast and accurate long-read alignment with Burrows-Wheeler transform, *Bioinformatics* **26**: 589-595.

Li, H., Handsaker, B., Wysoker, A., Fennell, T., Ruan, J., Homer, N., et al. (2009) The Sequence Alignment/Map format and SAMtools, *Bioinformatics* **25**: 2078-2079.

Martínez-García, E., and de Lorenzo, V. (2012) Transposon-based and plasmid-based genetic tools for editing genomes of gram-negative bacteria, *Methods Mol. Biol* **813**: 267-283.

Martínez-García, E., Nikel, P.I., Aparicio, T., and de Lorenzo, V. (2014) Pseudomonas 2.0: genetic upgrading of *P. putida* KT2440 as an enhanced host for heterologous gene expression, *Microb. Cell Fact.* **13**: 159.

Nikel, P.I., Chavarría, M., Fuhrer, T., Sauer, U., and de Lorenzo, V. (2015) *Pseudomonas putida* KT2440 Strain Metabolizes Glucose through a Cycle Formed by Enzymes of the Entner-Doudoroff, Embden-Meyerhof-Parnas, and Pentose Phosphate Pathways, *J. Biol. Chem.* **290**: 25920-25932.

Quinlan, A.R., and Hall, I.M. (2010) BEDTools: a flexible suite of utilities for comparing genomic features, *Bioinformatics* **26**: 841-842.

Tsuda, S., Jaffery, H., Doran, D., Hezwani, M., Robbins, P.J., Yoshida, M., and Cronin, L. (2015) Customizable 3D Printed 'Plug and Play' Millifluidic Devices for Programmable Fluidics, *PLOS ONE* **10**: e0141640.

Yoshida, M., Reyes, S.G., Tsuda, S., Horinouchi, T., Furusawa, C., and Cronin, L. (2017) Time-programmable drug dosing allows the manipulation, suppression and reversal of antibiotic drug resistance in vitro, *Nat. Commun.* **8**: 15589.
